# Melatonin is associated with cell-type-specific inflammatory and oxidative stress responses at the maternal-fetal interface

**DOI:** 10.3389/fimmu.2026.1897785

**Published:** 2026-09-22

**Authors:** Tyana T. Joseph, Toidi Adekambi, Kyra Brewer, Viviane Schuch, Nia Thompson, Dara Brena, Amber Dawning, Alaijah Bashi, Keturah Eaves, Cristina Wilson, Raimot Olanrewaju, Rana Chakraborty, Erica L. Johnson

**Affiliations:** 1 Microbiology, Biochemistry, and Immunology, Morehouse School of Medicine, Atlanta, GA, United States; 2 Department of Neurobiology, Morehouse School of Medicine, Atlanta, GA, United States; 3 Department of Obstetrics and Gynecology, Morehouse School of Medicine, Atlanta, GA, United States; 4 Department of Pediatrics, University of Miami, Miami, FL, United States

**Keywords:** decidual macrophages, maternal-fetal interface, melatonin, NLRP3, Nrf2, trophoblast cells

## Abstract

Melatonin regulates inflammation and oxidative stress at the placenta, but its effects on specific placental cell types remain unclear. This study investigated how melatonin influences inflammatory and oxidative stress responses, cytokine secretion, NRF2-associated antioxidant responses, and transcriptomic responses in JEG-3 trophoblast-like cells and primary decidual macrophages exposed to lipopolysaccharide (LPS). Melatonin reduced LPS-induced inflammatory cytokine production and intracellular reactive oxygen species accumulation, with pretreatment producing the strongest anti-inflammatory effects. Although NLRP3 transcript expression differed according to treatment sequence, melatonin was associated with reduced inflammatory cytokine secretion despite limited changes in total NLRP3 protein expression. Melatonin also differentially influenced antioxidant responses across placental cell types, with decidual macrophages exhibiting broader functional and transcriptional changes than JEG-3 trophoblast-like cells. Transcriptomic profiling revealed dose- and cell type-dependent effects, with decidual macrophages exhibiting altered expression of genes and pathways associated with inflammatory signaling, oxidative phosphorylation, and reactive oxygen species regulation. In contrast, JEG-3 trophoblast-like cells demonstrated limited transcriptional remodeling and more modest engagement of inflammatory signaling pathways than primary decidual macrophages. These findings suggest that melatonin differentially modulates inflammatory and oxidative stress responses across placental cell types at the maternal-fetal interface. Collectively, these data support the potential role of melatonin in promoting placental redox-inflammatory balance during inflammatory stress and provide a foundation for future studies in physiologically relevant models.

## 1 Introduction

The placenta is a highly specialized organ that sustains fetal growth and mediates communication between the mother and fetus (1–3). The placenta contains maternal decidual tissue and fetal chorionic components that network to regulate nutrient and gas exchange, hormone production, and immune balance throughout pregnancy (4, 5). Within this complex structure, fetal-derived trophoblasts, maternal decidual cells, and diverse immune cell populations interact to maintain equilibrium between immune tolerance and protection against inflammatory or infectious pathogens (6, 7). Among these immune populations, placental macrophages, including maternal decidual macrophages, play a central role in tissue homeostasis and host defense against infection and inflammation (8, 9). Their coordinated activity is essential for normal placental development and function (10–12).

Melatonin (N-acetyl-5-methoxytryptamine), widely known for its role in regulating circadian rhythms, is produced by the pineal gland and is also synthesized locally within the placenta during pregnancy (13). Trophoblast cells synthesize and secrete melatonin, reflecting both paracrine and autocrine activity to support placental function (14). Although maternal pineal melatonin readily crosses the placenta and supports fetal circadian rhythms, current evidence indicates this transplacental transfer primarily affects the fetus and does not elevate maternal systemic melatonin levels (15–18). Instead, elevated maternal melatonin concentrations observed during pregnancy reflect both increased maternal pineal output and placental melatonin production by trophoblasts, which contributes to the maternal circulation (19, 20).

Beyond its chronobiological role, melatonin has been reported to exhibit antioxidant and anti-inflammatory properties that protect placental tissue from oxidative stress and inflammatory injury (13, 21). Melatonin’s local production within the placenta stabilizes the microenvironment to support fetal development (22). By limiting oxidative stress, dampening inflammatory signaling, and preserving mitochondrial function, melatonin contributes to the normal homeostatic balance required for trophoblast survival, vascular development, and immune regulation at the maternal-fetal interface (23–27). When melatonin synthesis or signaling is disrupted, placental cells become more vulnerable to oxidative and inflammatory injury, a pattern linked to pregnancy complications such as preeclampsia, fetal growth restriction, preterm birth, and miscarriage (24, 27–29). These associations highlight melatonin’s role as an important contributor to placental resilience and emphasize the importance of understanding how different placental cell types respond to melatonin during inflammatory stress (24, 27, 30).

Melatonin’s biological effects depend on its concentration. At physiological and low nanomolar levels (1pM-10nM), melatonin acts mainly through its receptors MT1 and MT2 to modulate intracellular signaling pathways involved in immune and antioxidant regulation (31, 32). In the transition range (10nM to 100nM), melatonin exerts a combination of receptor-dependent and receptor-independent effects, including partial direct scavenging of reactive oxygen species (ROS) (33, 34). At higher pharmacologic or supraphysiological concentrations, such as the 1000nM dose used in this study, melatonin functions as a direct free-radical scavenger, neutralizing reactive oxygen species independent of receptor activation (31). This concentration-dependent distinction is particularly relevant to placental biology because receptor-mediated actions may modulate redox and immune responses, whereas higher concentrations provide more immediate ROS buffering during acute inflammatory stress (15, 24, 35, 36). Understanding how placental cells respond to both receptor-dependent and direct-scavenging actions of melatonin is essential for interpreting the dose-specific effects observed at the maternal-fetal interface. Accordingly, 1000nM was selected for subsequent mechanistic analyses because it consistently produced the greatest anti-inflammatory and antioxidant responses among the concentrations evaluated in this study and is a concentration commonly used in *in vitro* studies investigating the pharmacological effects of melatonin (26, 37, 38). The objective of using this concentration was not to reproduce physiological placental melatonin concentrations, but rather to assess the protective effects of melatonin under inflammatory conditions *in vitro*.

Melatonin has been widely reported to exert anti-inflammatory effects across numerous experimental models (39–42). One mechanism proposed to contribute to these protective effects is modulation of NLRP3-associated inflammatory responses. Despite growing evidence of melatonin’s anti-inflammatory effects, the mechanisms through which melatonin regulates inflammatory responses within the placenta remain incompletely understood. The NLRP3 inflammasome is a cytosolic multiprotein complex that drives the maturation and release of proinflammatory cytokines IL-18 and IL-1β (43, 44). Placental inflammation is influenced by NLRP3-associated inflammatory responses, although the contribution of this pathway varies depending on the inflammatory context. Excessive or prolonged activation of NLRP3 has been implicated in uncontrolled inflammation and placental pathology associated with several pregnancy complications, including preeclampsia, preterm birth, and microbial infection (45–48). Given melatonin’s role at the maternal-fetal interface and its capacity to influence inflammatory responses, it is important to better understand how melatonin modulates inflammatory pathways within the placenta.

Oxidative stress is a key upstream driver of inflammasome activity, and melatonin has been shown to counteract this process by activating the NRF2 pathway (49). NRF2 (nuclear factor erythroid 2- related factor 2) is a key transcriptional factor that regulates cellular redox homeostasis by inducing antioxidant and cytoprotective genes such as superoxide dismutase 2 (SOD2) (50–52). Under basal conditions, NRF2 is localized in the cytoplasm, where it is tightly bound to KEAP1, which targets NRF2 for proteasomal degradation (53). Oxidative stress disrupts this interaction, allowing NRF2 to translocate to the nucleus and bind antioxidant response elements (ARE) (54). Melatonin has been reported to modulate NRF2-associated antioxidant responses and promote nuclear localization of NRF2 in several experimental systems, thereby strengthening antioxidant defenses and reducing oxidative and inflammatory damage (55–57). Functional interactions between NRF2-associated antioxidant responses and NLRP3-associated inflammatory responses have been widely reported, with impaired NRF2 activity associated with enhanced oxidative stress and inflammatory responses (57). These observations suggest that melatonin may influence both antioxidant and inflammatory pathways at the maternal-fetal interface (58–60).

Despite growing evidence of melatonin’s antioxidant and anti-inflammatory functions, its cell type-specific effects in placental trophoblasts and macrophages remain poorly understood (24, 30). Therefore, this study investigated how melatonin modulates inflammatory and oxidative stress responses, cytokine secretion, NRF2-associated antioxidant responses, and transcriptomic responses in primary decidual macrophages and JEG-3 trophoblast-like cells exposed to inflammatory stimulation. JEG-3 is a choriocarcinoma-derived trophoblast-like cell line widely used as an *in vitro* model because it retains several trophoblast-associated characteristics, including hormone production and responsiveness to inflammatory stimuli. However, as a transformed cell line, JEG-3 cells do not fully recapitulate the phenotype or functional complexity of primary trophoblasts. Accordingly, findings obtained using this model should be interpreted within the context of its inherent biological limitations. This study provides insight into the cell type-specific modulation of inflammatory and oxidative stress responses by melatonin at the maternal-fetal interface and contributes to our understanding of its potential immunomodulatory role during placental inflammation. These findings establish a foundation for future studies using primary trophoblasts, placental explants, and *in vivo* models to further define the role of melatonin during placental inflammation.

## 2 Methods

### 2.1 Study approval

After obtaining written informed consent from healthy pregnant individuals (>18 years old and hepatitis B, HIV-1 seronegative), placentae (>37 weeks’ gestation) were obtained after cesarean delivery from Grady Memorial Hospital in Atlanta, Georgia. All samples were anonymized before processing and handling by laboratory personnel. Study approval was granted by the Morehouse School of Medicine Institutional Review Board (1964407–8) and the Grady Oversight Committee.

### 2.2 Dissection of placenta and decidual macrophage isolation

Decidual macrophages from term placentae were isolated from the maternal side of the placenta as described previously (61). The decidual basalis was dissected from the placenta. The decidua basalis was then washed extensively with ice-cold Phosphate-Buffered Saline (PBS) (Corning Cellgro, Corning, NY, USA) to minimize blood contamination and minced for tissue digestion to obtain decidual macrophages. Isolation of decidual macrophages consisted of one tissue digestion: 1mg/ml Collagenase IV(Worthington Biochemicals, Lakewood, NJ, USA), 0.25mg/ml DNase I (Thermo Scientific, Waltham, MA, USA), 5% HEPES (Corning Cellgro, Corning, NY, USA), 47.5% Roswell Park Memorial Institute (RPMI) medium(Corning), and 47.5% complete RPMI medium(Corning) (10% Fetal Bovine Serum (FBS) (Optima, Atlanta Biologics, Atlanta, GA, USA), 1% penicillin-streptomycin (pen-strep) (Corning), 2mM L-Glutamine (Gibco, Waltham, MA, USA), and 2.52% HEPES (Corning)) for 1 hour at 37°C in the New Brunswick Scientific Excella E24 Incubator Shaker. The digested tissue was passed through gauze and a sequence of cell strainers (100μm, 70μm, and 40μm) (BD-Falcon Biosciences, San Jose, CA, USA). The mononuclear cell population was isolated using density gradient centrifugation on Ficoll-Paque Plus (Sigma-Aldrich, St. Louis, MO, USA). Cell sorting was completed using the CD14^+^ microbead magnetic system (Miltenyi Biotech, Bergisch Gladbach, Germany) according to the manufacturer’s protocols. After isolation, decidual macrophages were cultured in complete RPMI medium: 1x RPMI medium (Corning), 10% FBS (Optima), 1% pen-strep (Corning), 2mM L-Glutamine (Gibco), and 2.52% HEPES at 37°C and 5% CO_2_.

### 2.3 Cell culture

JEG-3 trophoblast-like cells (ATCC HTB-36, Manassas, VA, US), a choriocarcinoma-derived cell line, were grown and maintained according to ATCC instructions: 500 mL Eagle’s Minimum Essential Medium (EMEM) (ATCC 30-2003) supplemented with 10% FBS (Optima), and 1% pen-strep (Corning), at 37°C with 5% CO_2_. JEG-3 cells were detached using trypsin (Corning), counted, and then seeded based on the downstream assay.

### 2.4 Cell culture treatments

JEG-3 and decidual macrophages were treated with lipopolysaccharide (LPS) 5ug/ml (Invitrogen, Carlsbad, CA, USA) for 4 hours, then treated with various concentrations of melatonin (1nM, 10nM, 100nM, and 1000nM) (MP Biomedicals, Santa Ana, CA, USA) for 24 hours or 48 hours. For post-treatment experiments, cells were treated with various concentrations of melatonin (1nM, 10nM, 100nM, and 1000nM) for 24 hours or 48 hours, then treated with LPS for 4 hours. Cell viability was assessed using the CYQUANT ™ MTT Cell Viability Assay Kit (Invitrogen) according to the manufacturer’s instructions.

### 2.5 Real-time polymerase chain reaction

JEG-3 cells were seeded/treated at 5x10^5^ cells/ml and decidual macrophages at 1x10^6^ cells/ml in 24-well plates and treated for 24h as previously described. Messenger RNA (mRNA) was isolated from cells using the RNeasy Mini Kit (Qiagen, Venlo, Netherlands). The total RNA concentration was measured using a Nanodrop spectrophotometer, with 200ng of RNA used for complementary DNA (cDNA) reverse transcription. cDNA was transcribed using the iScript™ gDNA Clear cDNA Synthesis kit (Bio-Rad, Hercules, CA, USA). Gene expression was conducted by SYBR Green with appropriate primers (NRF2, NFκB, NLRP3, and SOD2). Reactions occurred in duplicates using the CFX OPUS 96 Real-Time PCR System. Relative expression levels were measured by using the 2^-ΔΔCt^ formula. The endogenous housekeeping gene, β-actin, was used for normalization. The gene expression data are portrayed as fold changes relative to time-matched experimental groups.

### 2.6 Western blot

JEG-3 cells were seeded at 5x10^5^ cells/ml and decidual macrophages at 1x10^6^ cells/ml in 12-well plates and treated for 48h as previously described. Total protein was isolated from lysates of primary decidual macrophages and JEG-3 cells using Pierce™ RIPA lysis buffer (Thermo Scientific) supplemented with 1% Protease Inhibitor Cocktail Kit (Thermo Scientific) and 1% Halt™ Phosphatase Inhibitor Cocktail (Thermo Scientific). The concentrations of the proteins were determined by Pierce™ BCA Protein Assay Kit (Thermo Scientific). Based on volume, Bolt™ Sample Reducing Agent (10X) (Invitrogen) and NuPage® LDS Sample Buffer (4X) were added to the protein, and the protein was denatured for 10 minutes. Gel electrophoresis was completed using NuPage™ Bis-Tris Mini Protein Gel, 10%-12%. The gel was then transferred onto a polyvinylidene fluoride (PVDF) membrane (Invitrogen) and blocked for 1 hour with 5% Bovine Serum Albumin (BSA) in 1X Tris-buffered saline and Tween-20 (TBST). Following protein transfer, PVDF membranes were horizontally sectioned according to the expected molecular weights of the target proteins. Membrane sections were probed with the indicated primary antibodies, and where appropriate, the same membrane section was sequentially stripped using Restore™ Western Blot Stripping Buffer Thermo Scientific) and reprobed for additional protein targets. Membranes were probed with primary antibodies overnight at 4°C: rabbit-anti-NLRP3 (13158S), rabbit-anti-NRF2 (PA5-27882), rabbit-anti-pNRF2 (bs-23531R), and rabbit-anti-GAPDH (D16H11) [Cell Signaling Technology, Danvers, MA, USA; Thermo Fisher Scientific, Waltham, MA, USA; Bioss, Woburn, MA, USA]. The following day, the membrane was washed three times with TBS-T and incubated with goat anti-rabbit, HRP-linked secondary antibodies (7074S) for 1 hour. The membrane was washed 3 times with TBS-T and developed using Pierce™ Western Blot Signal Enhancer (Thermo Scientific). Band intensities for NLRP3, NRF2, and pNRF2 were quantified using the iBright 1500 Imaging System and normalized to GAPDH. For NRF2 analyses, pNRF2/NRF2 ratios were subsequently calculated for statistical comparisons. Uncropped, high-resolution Western blot images are provided in the **Supplementary Material**.

### 2.7 Enzyme-linked immunosorbent assay (ELISA)

Interleukin-1β (IL-1β), IL-6, and IL-10 concentrations in the supernatants of treated JEG-3 trophoblast-like cells, primary decidual macrophages, and their corresponding controls were determined using ELISA MAX Deluxe Set Human Kits (BioLegend, San Diego, CA, USA) according to the manufacturer’s instructions. Interleukin-18 (IL-18) concentrations were determined using the Human IL-18 DuoSet ELISA kit (R&D Systems, Minneapolis, MN, USA) according to the manufacturer’s instructions. All samples were analyzed in duplicate. Plates were read using a BioTek Synergy H1 microplate reader, and cytokine concentrations were calculated by comparison with standard curves. Samples with cytokine concentrations below the assay detection limit were recorded as undetectable and were not extrapolated beyond the standard curve.

### 2.8 Immunofluorescence

JEG-3 cells were seeded at 2.5x10^5^ cells/ml on Neuvitro™ coverslips pre-coated with collagen (Fischer Scientific). Decidual macrophages were seeded at 1.5x10^6 cells/ml on Neuvitro coverslips pre-coated with poly-d-lysine. The JEG-3 cells and decidual macrophages were treated (No Treatment, Vehicle, LPS, 1000nM MEL, LPS + 1000nM MEL, and 1000nM MEL+LPS) for 24 hours as previously described. After treatment incubation, cells were washed with PBS and fixed with 2ml of ice-cold 4% Paraformaldehyde in PBS (USB Corporation, Cleveland, OH, USA) for 10 minutes at room temperature. Fixed cells were then permeabilized with 0.1% TritonX-100 (Thermo Scientific) in PBS for 10 minutes at room temperature and blocked with 3% BSA in PBS for 1 hour at room temperature on a rocker. Fixed cells were probed with the primary antibody rabbit-anti-pNRF2 (bs-23531R) overnight at 4 °C. The following day, the fixed cells were washed three times with PBS and incubated with goat anti-rabbit, IgG (Heavy Chain), Superclonal™ Recombinant Secondary Antibody, Alexa Fluor™ 488 (Thermo Scientific) for 2 hours at room temperature on a rocker. Cells were washed three times and stained with DAPI (Invitrogen) for 5 minutes at room temperature. Cells were washed three times and imaged using the Eclipse Ts2 (Nikon) microscope.

### 2.9 Detection of reactive oxygen species

For ROS quantification, JEG-3 cells were seeded at 2.5x10^4^ cells/ml, and decidual macrophage cells were seeded at 1x10^5^ cells/ml in triplicate in 96-well plates, and treated (No Treatment, Vehicle, LPS, 1000nM MEL, LPS + 1000nM MEL, and 1000nM MEL+LPS) for 24h as previously described. According to the manufacturer’s protocols, intracellular ROS were detected and measured via DCFH-DA fluorescent probe (Invitrogen). ROS levels were measured and detected using a fluorescent microscope (Nikon Eclipse Ts2) and quantified using CellProfiler software.

For fluorescent images only, JEG-3 cells were seeded at 1x10^5^ cells/ml, and decidual macrophage cells were seeded at 8x10^5^ cells/ml in duplicates in 24-well plates, and treated (No Treatment, Vehicle, LPS, 1000nM MEL, LPS + 1000nM MEL, and 1000nM MEL+LPS) for 24h. According to the manufacturer’s protocols, intracellular ROS were detected and measured via DCFH-DA fluorescent probe (Invitrogen). Cells were washed three times and stained with Cell Signaling Hoechst 33342 for 5 minutes at room temperature. Cells were washed three times and imaged using the Eclipse Ts2 (Nikon) microscope.

### 2.10 Detection of superoxide dismutase

Superoxide dismutase (SOD) activity in treated JEG-3 trophoblast-like cells, primary decidual macrophages, and their corresponding controls was determined using the Superoxide Dismutase Assay Kit (Cayman Chemical, Ann Arbor, MI, USA) according to the manufacturer's instructions. Cell lysates were prepared following treatment, and samples were analyzed in duplicate. The assay utilizes a tetrazolium salt for detection of superoxide radicals generated by xanthine oxidase and hypoxanthine. Absorbance was measured at 450 nm using a BioTek Synergy H1 microplate reader. SOD activity was calculated according to the manufacturer's instructions and expressed relative to the corresponding control conditions. Samples with concentrations below the assay detection limit were recorded as undetectable and were not extrapolated beyond the standard curve.

### 2.11 RNA-seq analysis

JEG-3 cells were seeded/treated at 5x10^5^ cells/ml and decidual macrophages at 1x10^6^ cells/ml in duplicate in 24-well plates and treated for 24h as previously described. The treatment of the cells was as follows: No Treatment, Vehicle, 1nM melatonin, and 1000nM melatonin. RNA-seq analyses were performed under basal treatment conditions and did not include LPS stimulation. Total RNA was extracted from treated JEG-3 cells and decidual macrophages using the RNeasy Mini Kit. NanoDrop assessed RNA purity and concentration. RNA integrity was confirmed by Bioanalyzer (RIN ≥ 7). mRNA libraries were prepared using the MGIEasy RNA Directional Library Prep Kit, which includes poly-A selection and strand-specific synthesis. Libraries were sequenced by BGI Genomics on the BGISEQ-500/MGISEQ platform (PE100), generating ~3–5 Gb per sample. Low-quality reads, adapters, and reads with >5% ambiguous bases were removed.

Clean reads were aligned to GRCh38 using STAR (v2.7) and quantified with featureCounts. Only uniquely mapped, properly paired reads were retained. Genes with extremely low counts were excluded. Differential expression analysis was performed in DESeq2 using the median-ratio normalization method and a negative binomial model. Wald tests were used to calculate log_2_ fold changes and adjusted p-values (Benjamini–Hochberg). Log_2_FC shrinkage used the apeglm method. Significant genes were defined as false discovery rate (FDR)≤0.10. A variance-stabilizing transformation (VST) was applied for PCA to evaluate clustering, major variance drivers, and sample quality. Gene set enrichment analysis was performed using fgsea with MSigDB Hallmark gene sets (category H). Genes were ranked by the DESeq2 Wald statistic. Pathways with FDR ≤ 0.05 were visualized in R using ggplot2 and heatmap. Differential expressed genes with FDR≤ 0.05 were also visualized using volcano plots.

### 2.12 Statistics

Statistical analyses were performed using GraphPad Prism 10 (GraphPad Software, Boston, MA, USA). Depending on data distribution and experimental design, data were analyzed using parametric or non-parametric tests, including one-way ANOVA or repeated-measures one-way ANOVA followed by the appropriate multiple-comparisons tests. The specific statistical test used for each experiment is indicated in the corresponding figure legend. Statistical significance was defined as p ≤ 0.05.

### 2.13 Superoxide dismutase activity assay

Superoxide dismutase (SOD) activity in treated primary human decidual macrophages and their corresponding controls was determined using the Superoxide Dismutase Assay Kit (Cayman Chemical, Ann Arbor, MI, USA) according to the manufacturer's instructions. Cell lysates were prepared following treatment, and samples were analyzed in duplicate. The assay utilizes a tetrazolium salt for detection of superoxide radicals generated by xanthine oxidase and hypoxanthine. Absorbance was measured at 450 nm using a BioTek Synergy H1 microplate reader. SOD activity was calculated according to the manufacturer's instructions. Samples with SOD activity below the assay detection limit were recorded as undetectable and were not extrapolated beyond the standard curve.

## 3 Results

### 3.1 Treatment conditions do not alter cell viability

To determine whether the observed changes in inflammatory and oxidative stress responses were attributable to cytotoxicity, cell viability was assessed following LPS and melatonin treatment. No significant differences in cell viability were observed in either JEG-3 cells or primary decidual macrophages under the experimental conditions used (**Supplementary Figure 1**).

### 3.2 Melatonin differentially modulated inflammatory gene expression in decidual macrophages and JEG-3 cells

To determine whether melatonin modulates inflammatory gene expression during inflammatory stimulation, NLRP3 and NFκB mRNA expression were measured in JEG-3 cells and primary decidual macrophages following LPS stimulation and melatonin treatment. Because these analyses were designed to evaluate whether melatonin modified LPS-induced inflammatory gene expression, LPS-stimulated melatonin-treated groups were compared directly with the corresponding LPS-treated control. Accordingly, these analyses were intended to assess melatonin-associated modulation of inflammatory gene expression under inflammatory conditions rather than the independent effects of melatonin under basal conditions.

In JEG-3 cells, melatonin (1nM, 10nM, 100nM, 1000nM) administered either before or after LPS stimulation did not significantly alter NLRP3 mRNA expression compared with LPS-treated cells (**Figure 1a**). In primary decidual macrophages, LPS treatment significantly increased NLRP3 mRNA expression compared with the vehicle control (**Figure 1a**). Similarly, 1000nM melatonin administered after LPS stimulation and 1nM melatonin administered before LPS stimulation remained significantly elevated relative to the vehicle control. In contrast, pretreatment with 10nM, 100nM, and 1000nM melatonin significantly reduced NLRP3 mRNA expression compared with LPS-treated cells.

**Figure 1:**
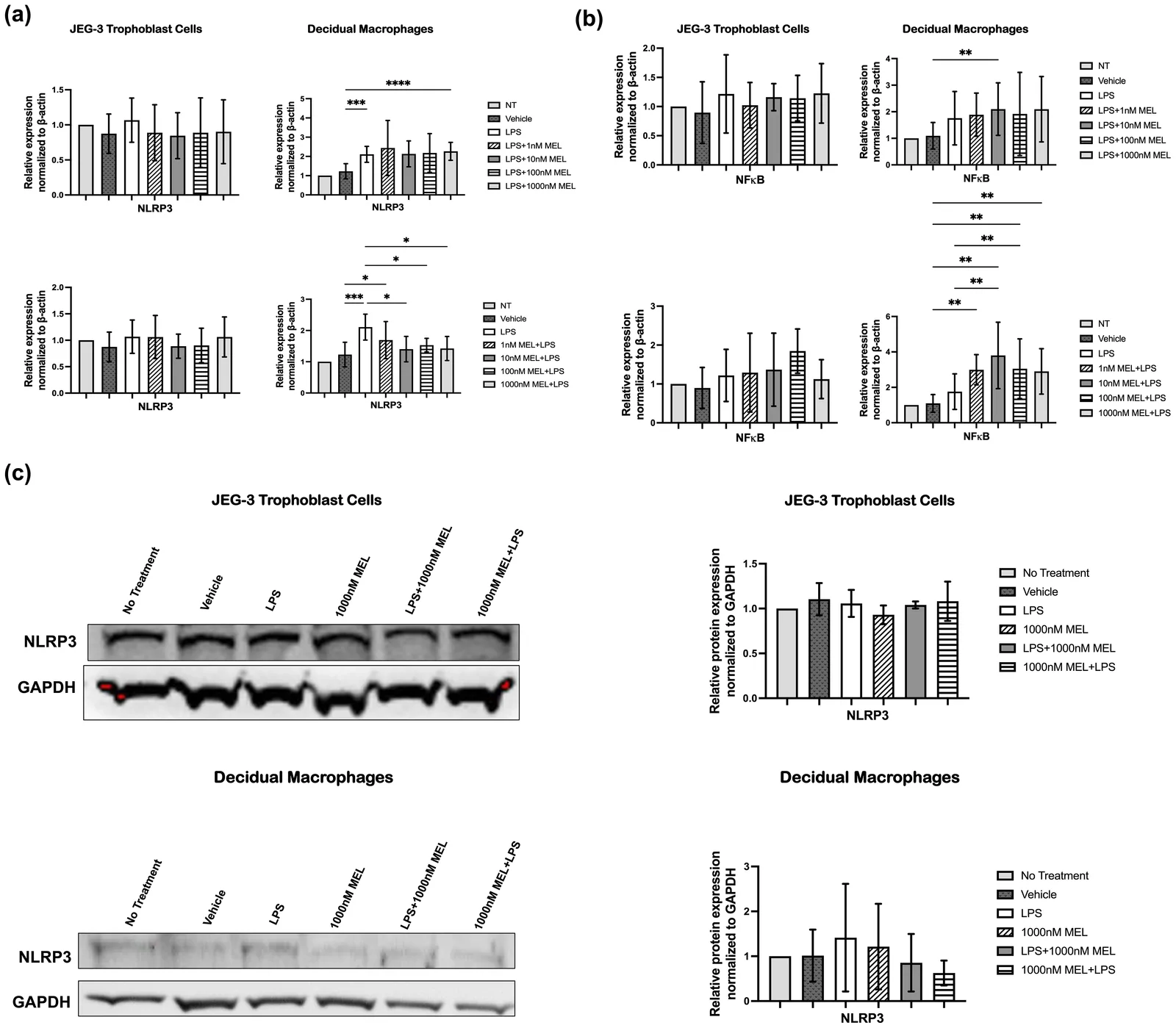
Melatonin differentially modulates inflammatory gene expression but does not significantly alter NLRP3 protein expression in placental cells. **(a)** JEG-3 trophoblast-like cells were cultured, and primary human decidual macrophages were isolated and treated with LPS (5μg/ml), LPS for 4h followed by melatonin (1-1000nM, 24h), melatonin for 24h followed by LPS for 4h, or vehicle. NLRP3 transcript levels were measured by qRT-PCR and expressed as ΔCT values normalized to β-actin. Data are presented as the mean ± SD (n= 5 independent biological donors). Technical duplicates were performed for each donor. For JEG-3 data, statistical analyses were performed using the Kruskal-Wallis test followed by Dunn’s multiple-comparisons test, whereas decidual macrophage data were analyzed using repeated-measures one-way ANOVA followed by Šídák’s multiple-comparisons test. Brackets denote the specific pairwise shown. Statistical Significance is indicated as: ****p≤0.0001, ***p≤0.001, **p≤0.01, and *p≤0.05. **(b)** NFκB mRNA transcript levels were measured by qRT-PCR and expressed as ΔCT normalized to β-actin. Data are presented as the mean ± SD (JEG-3: n= 4 independent biological donors; Decidual macrophages: n= 5 independent biological donors). Technical duplicates were performed for each donor. For JEG-3 data, statistical analyses were performed using the Kruskal-Wallis test followed by Dunn’s multiple-comparisons test, whereas decidual macrophage data were analyzed using repeated-measures one-way ANOVA followed by Šídák’s multiple-comparisons test. Brackets denote the specific pairwise shown. Statistical Significance is indicated as: ****p≤0.0001, ***p≤0.001, **p≤0.01, and *p≤0.05. **(c)** JEG-3 cells and primary decidual macrophages were treated with LPS (5 μg/mL), melatonin (1000 nM), LPS followed by melatonin, melatonin followed by LPS, or vehicle. Protein lysates were separated by SDS-PAGE, transferred to PVDF membranes, and immunoblotted for NLRP3 and GAPDH. Following protein transfer, PVDF membranes were horizontally sectioned near the 50 kDa molecular weight marker to permit independent probing of the upper membrane section for NLRP3 and the lower membrane section for GAPDH. Where applicable, membrane sections were sequentially stripped and reprobed with the indicated antibodies. Band intensities for NLRP3 were quantified using the iBright 1500 Imaging System, normalized to GAPDH, and are presented as the mean ± SD from five donors. Statistical analyses were performed using one-way ANOVA with Tukey’s *post hoc* test. Representative bands are shown. Full, uncropped Western blot membranes with molecular weight markers are provided in **Supplementary Figure 1**. Statistical analyses were performed using one-way ANOVA with Tukey’s post-test. Brackets denote the specific pairwise shown. Statistical significance is indicated as: ****p≤0.0001, ***p≤0.001, **p≤0.01, and *p≤0.05.

NFκB transcript expression was also evaluated as an additional marker of inflammatory gene expression (**Figure 1b**). In JEG-3 cells, melatonin treatment administered either before or after LPS stimulation did not significantly alter NFκB mRNA expression relative to the vehicle control. In primary decidual macrophages, NFκB transcript expression was significantly increased in the 10nM melatonin group compared with the vehicle when melatonin was administered after LPS stimulation. When melatonin was administered before LPS stimulation, NFκB transcript expression was significantly increased at 1nM, 10nM, 100nM, and 1000nM melatonin compared with vehicle. In addition, pretreatment with 10nM and 100nM melatonin significantly increased NFκB transcript expression compared with LPS-treated cells.

Overall, these findings indicate that inflammatory gene expression responded differently to melatonin treatment in primary decidual macrophages and JEG-3 trophoblast-like cells. Treatment-sequence-dependent changes in NLRP3 and NFκB transcript expression were observed primarily in decidual macrophages, suggesting that maternal immune cells were more transcriptionally responsive to melatonin under inflammatory conditions.

### 3.3 Melatonin does not significantly alter NLRP3 protein expression in placental cells during LPS stimulation

Western blot analysis was performed to determine whether changes in NLRP3 mRNA expression were reflected at the protein level (**Figure 1c**). In JEG-3 cells, NLRP3 protein was detected across all treatment groups. Neither LPS stimulation nor melatonin treatment, regardless of treatment sequence, produced a statistically significant change in NLRP3 protein expression. Similarly, in decidual macrophages, NLRP3 protein was detectable in vehicle-treated cells and LPS-exposed cells. Melatonin treatment administered either before or after LPS stimulation did not significantly alter NLRP3 protein expression compared with either the vehicle control or LPS-treated cells. Although treatment-sequence-dependent changes in NLRP3 mRNA expression were observed in decidual macrophages, these transcriptional changes were not accompanied by corresponding changes in total NLRP3 protein expression under the experimental conditions examined.

### 3.4 Melatonin modulates LPS-induced cytokine secretion in decidual macrophages and JEG-3 trophoblast-like cells

LPS stimulation increased inflammatory cytokine secretion in both placental cell types, although the magnitude of the response differed between JEG-3-trophoblast-like cells and primary decidual macrophages. To determine whether melatonin modulated inflammatory cytokine production during LPS stimulation, IL-1β, IL-6, IL-10, and IL-18 secretion were quantified following melatonin treatment.

In JEG-3 cells, vehicle-treated cells exhibited significantly lower IL-1β secretion than untreated controls (**Figure 2a**). Although the mechanism underlying this unexpected observation is unclear, all subsequent treatment effects were interpreted relative to the corresponding vehicle control. LPS stimulation significantly increased IL-1β secretion compared with the vehicle control. Treatment with 1000nM melatonin alone also reduced IL-1β secretion compared with LPS-treated cells. Similarly, melatonin pretreatment before LPS stimulation significantly increased IL-1β secretion compared with the vehicle control. IL-6, IL-10 and IL-18 concentrations remained below the assay detection limit under all treatment conditions. Because these cytokines were undetectable in both unstimulated and LPS-treated JEG-3 cultures, it was not possible to determine whether this reflected limited cytokine production by JEG-3 trophoblast-like cells under the present experimental conditions or concentrations below the analytical sensitivity of the assay.

**Figure 2:**
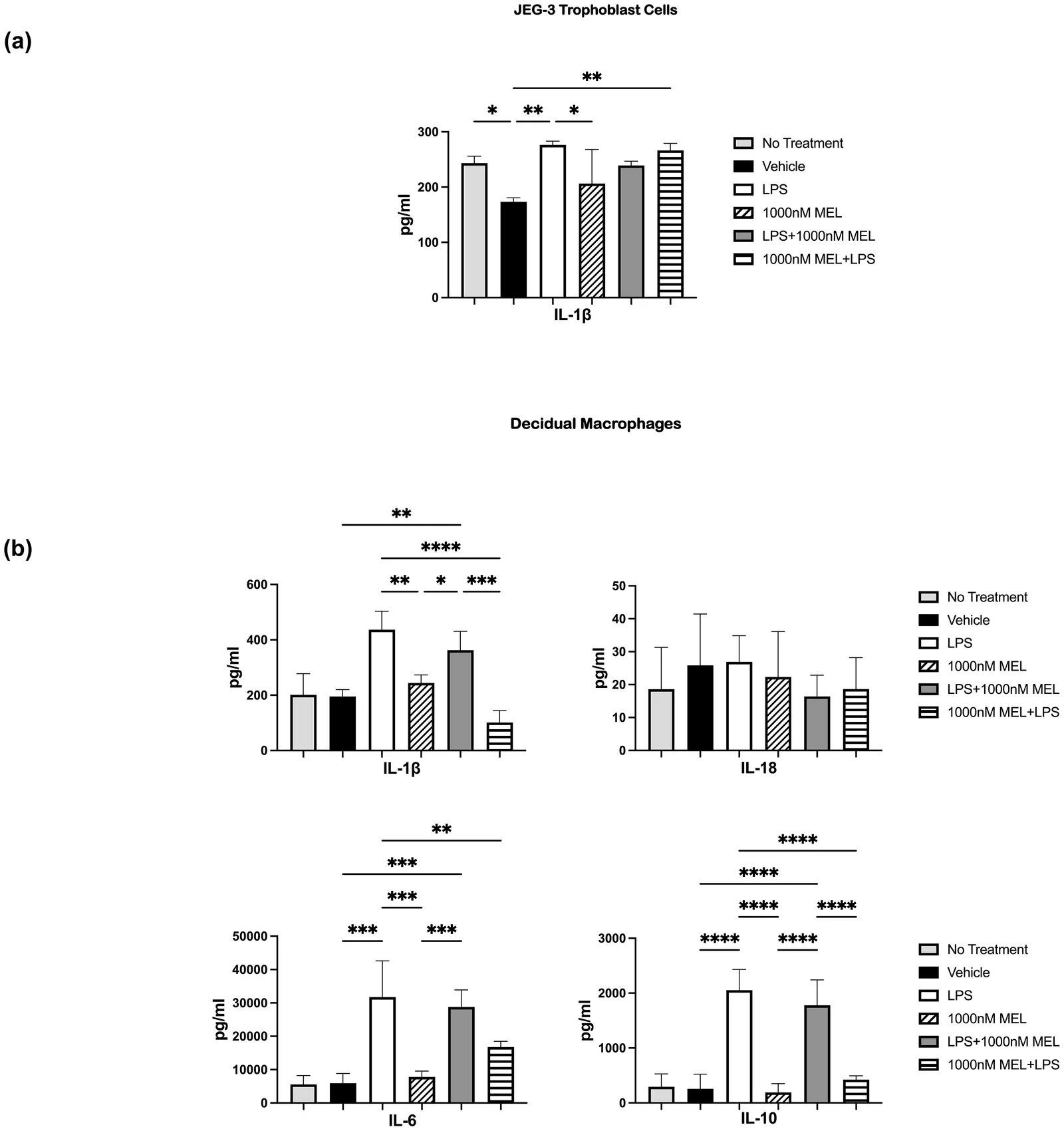
Melatonin reduces LPS-induced cytokine secretion in decidual macrophages and JEG-3 cells. **(a)** JEG-3 cells were treated with LPS (5μg/ml), melatonin (1000nM), LPS followed by melatonin, melatonin followed by LPS, or vehicle. IL-1β levels in culture supernatants were quantified after 24h and are shown as pg/ml. Data are presented as the mean ± SD (n= 3 independent biological donors). Technical duplicates were performed for each donor and averaged prior to statistical analysis. Statistical analyses were performed using one-way ANOVA followed by Šídák’s multiple-comparisons test. Brackets denote the specific pairwise shown. Statistical significance is indicated as: ****p≤0.0001, ***p≤0.001, **p≤0.01, and *p≤0.05. **(b)** Decidual macrophages were treated with LPS (5μg/ml), melatonin (1000nM), LPS followed by melatonin, melatonin followed by LPS, or vehicle. IL-1β, IL-6, IL-10, and IL-18 were measured in supernatants by ELISA after 24h and are reported as pg/ml. Data are presented as the mean ± SD of three independent biological donors for IL-1β, IL-6, and IL-18, with technical duplicates performed for each donor. Data are presented as the mean ± SD of four independent biological donors for IL-10, with technical duplicates performed for each donor. Statistical analyses were performed using one-way ANOVA followed by Holm-Šídák’s multiple-comparisons test. Brackets denote the specific pairwise shown. Statistical significance is indicated as: ****p≤0.0001, ***p≤0.001, **p≤0.01, and *p≤0.05.

In primary decidual macrophages, LPS significantly increased IL-6 and IL-10 secretion compared with vehicle-treated cells (**Figure 2b**). Treatment with 1000nM melatonin alone significantly reduced three cytokines (IL-1β, IL-6, and IL-10) compared to LPS-treated cells. Likewise, melatonin pretreatment before LPS significantly decreased IL-1β, IL-6, and IL-10 secretion relative to LPS alone. When melatonin was administered after LPS stimulation, cytokine concentrations remained significantly higher than melatonin alone for IL-1β, IL-6, and IL-10, suggesting that inflammatory cytokine production remained elevated following prior LPS stimulation under the experimental conditions used. Interestingly, treatment sequence influenced select inflammatory responses. Melatonin pretreatment (1000nM MEL + LPS) resulted in significantly lower IL-1β and IL-10 secretion than melatonin administered following LPS stimulation (LPS + 1000nM MEL), whereas IL-6 did not differ significantly between the two treatment sequences. These findings suggest that the order of melatonin administration influenced the magnitude of select cytokine responses under the experimental conditions used. IL-18 secretion was also evaluated as an additional marker associated with inflammasome activation. Although IL-18 was detectable in primary decidual macrophages, no statistically significant differences were observed among treatment groups.

Collectively, these findings indicate that melatonin was associated with attenuation of LPS-induced cytokine secretion in both placental cell types, with the greatest anti-inflammatory effects observed when melatonin was administered prior to inflammatory stimulation. Although IL-18 secretion was not significantly altered under the experimental conditions examined, the more pronounced responses observed in decidual macrophages further support cell type-specific differences in melatonin responsiveness during inflammatory challenge.

### 3.5 Melatonin influences NRF2 expression, pNRF2 protein levels, and pNRF2 nuclear localization in placental cells during inflammatory

To investigate whether melatonin modulated antioxidant defense through the NRF2 signaling pathway, NRF2 mRNA expression, pNRF2 protein expression, and pNRF2 cellular localization were evaluated in JEG-3 cells and primary decidual macrophages.

In JEG-3 cells, melatonin treatment administered either before or after LPS stimulation did not significantly alter NRF2 mRNA expression relative to the vehicle control (**Figure 3a**). Similarly, no significant differences were observed between LPS-treated cells and melatonin-treated groups under either treatment sequence. In primary decidual macrophages, several melatonin-treated groups exhibited significantly higher NRF2 mRNA expression compared with the vehicle control. Specifically, NRF2 transcript expression was significantly increased following 10nM and 100nM melatonin administered after LPS stimulation, as well as following 1nM, 10nM, and 1000nM melatonin pretreatment prior to LPS stimulation. No significant differences were detected between LPS-treated cells and any melatonin-treated group.

**Figure 3:**
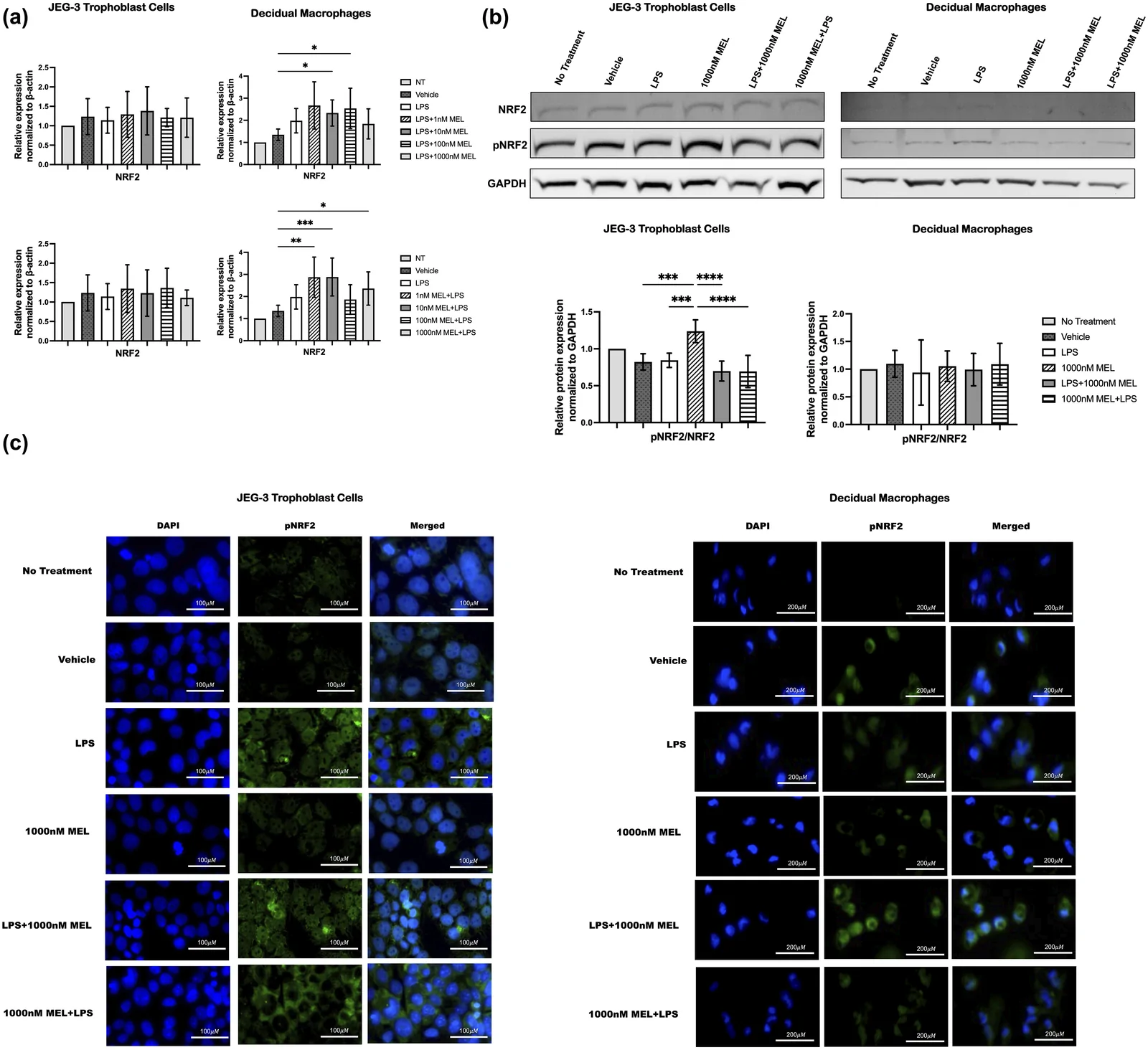
Melatonin modulates NRF2- associated responses and pNRF2 Localization in JEG-3 trophoblast-like cells and decidual macrophages. **(a)** JEG-3 trophoblast-like cells and primary human decidual macrophages were treated with LPS (5μg/ml), LPS for 4h followed by melatonin (1-1000nM, 24h), melatonin for 24h followed by LPS for 4h, or vehicle. NRF2 transcript levels were measured by qRT-PCR and expressed as ΔCT values normalized to β-actin. Data are presented as the mean ± SD (JEG-3: n= 4 independent biological donors; Decidual macrophages: n= 5 independent biological donors). Technical duplicates were performed for each donor. For JEG-3 data, statistical analyses were performed using the Kruskal-Wallis test followed by Dunn’s multiple-comparisons test, whereas decidual macrophage data were analyzed using repeated-measures one-way ANOVA followed by Šídák’s multiple-comparisons test. Brackets denote the specific pairwise shown. Statistical significance is indicated as: ****p≤0.0001, ***p≤0.001, **p≤0.01, and *p≤0.05. **(b)** JEG-3 cells and primary decidual macrophages were treated with LPS (5 μg/mL), melatonin (1000 nM), LPS followed by melatonin, melatonin followed by LPS, or vehicle. Protein lysates were separated by SDS-PAGE, transferred to PVDF membranes, and immunoblotted for NRF2, pNRF2, and GAPDH. Following protein transfer, PVDF membranes were horizontally sectioned near the 50 kDa molecular weight marker to permit independent probing of the upper membrane section for NRF2 and pNRF2 and the lower membrane section for GAPDH. Where applicable, membrane sections were sequentially stripped and reprobed with the indicated antibodies. Band intensities for NRF2 and pNRF2 were quantified using the iBright 1500 Imaging System, normalized to GAPDH, and are presented as the mean ± SD from five donors. Statistical analyses were performed using one-way ANOVA with Tukey’s *post hoc* test. Representative bands are shown. Full, uncropped Western blot membranes with molecular weight markers are provided in **Supplementary Figures 2** and **S3**. **(c)** Immunofluorescence staining of pNRF2 in JEG-3 and decidual macrophages following treatment with LPS (5μg/ml), melatonin (1000nM), LPS followed by melatonin, melatonin followed by LPS, or vehicle. DAPI (blue) marks nuclei, and pNRF2 staining is shown in green. JEG-3 magnification, 20x; decidual macrophages, 20x.

Western blot analysis was performed to determine whether transcriptional changes in NRF2 were accompanied by corresponding changes in pNRF2 protein expression (**Figure 3b**). In JEG-3 cells, treatment with 1000nM melatonin alone significantly increased the pNRF2/NRF2 protein ratio compared with the vehicle control and LPS-treated cells. In contrast, pNRF2/NRF2 expression was significantly reduced when melatonin was administered before or after LPS stimulation compared with melatonin treatment alone. No significant differences were observed between vehicle-treated cells, LPS-treated cells, and the melatonin pretreatment or post-treatment groups. In primary decidual macrophages, no significant differences in pNRF2/NRF2 protein expression were detected among treatment groups.

Immunofluorescence staining was subsequently performed to evaluate the subcellular localization of pNRF2 (**Figure 3c**). In JEG-3 cells and decidual macrophages, LPS treatment was associated with predominantly cytoplasmic pNRF2 staining relative to untreated controls. When melatonin was added after LPS stimulation, pNRF2 staining appeared more pronounced in the perinuclear and nuclear regions compared to LPS alone. In JEG-3 cells pretreated with melatonin before LPS, pNRF2 staining remained largely cytoplasmic, with limited apparent nuclear enrichment. A similar predominantly cytoplasmic distribution was observed in decidual macrophages under pretreatment conditions.

Collectively, these findings demonstrate distinct cell-type-specific patterns of NRF2 regulation at the maternal-fetal interface. While changes in NRF2 mRNA expression were more evident in primary decidual macrophages, alterations in pNRF2 protein expression were primarily observed in JEG-3 trophoblast-like cells. These findings indicate that transcriptional responses were not uniformly reflected at the protein level under the experimental conditions examined. Immunofluorescence further suggested that the timing of melatonin exposure influences pNRF2 cellular localization during inflammatory stimulation.

### 3.6 Melatonin mitigates LPS-induced oxidative stress and induces SOD2 mRNA expression

Intracellular reactive oxygen species (ROS) production was evaluated using DCFH-DA fluorescence following 24 hours of treatment (**Figure 4a**). In both JEG-3 cells and primary decidual macrophages, LPS stimulation significantly increased intracellular ROS levels compared with the vehicle control. Treatment with 1000nM melatonin alone exhibited significantly lower ROS levels than LPS-treated cells in both cell types. Furthermore, administration of 1000nM melatonin either before or after LPS stimulation significantly reduced ROS generation compared with LPS alone. Representative fluorescence images demonstrated increased DCF fluorescence following LPS exposure, whereas melatonin treatment under both treatment sequences was associated with visibly reduced fluorescence intensity. Quantification of fluorescence confirmed that melatonin significantly attenuated LPS-induced oxidative stress in both JEG-3 cells and primary decidual macrophages (**Figure 4b**).

**Figure 4:**
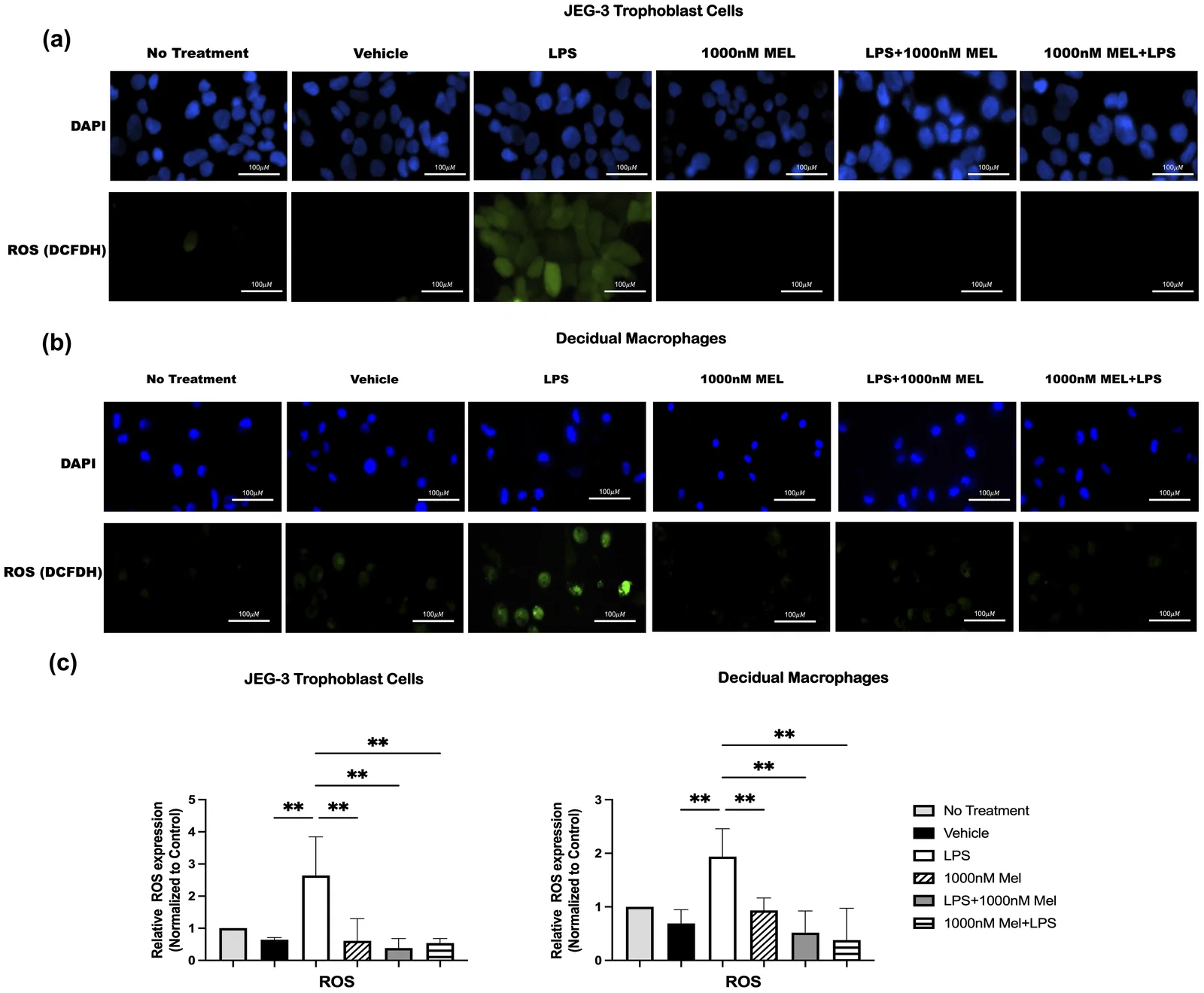
Melatonin mitigates LPS-induced oxidative stress. **(a)** Cellular ROS in JEG-3 and decidual macrophages was determined using DCFH-DA fluorescent probe following treatment with LPS (5μg/ml), melatonin (1000nM), LPS followed by melatonin, melatonin followed by LPS, or vehicle. DAPI (blue) marks nuclei, and ROS/DCFH-DA staining is shown in green. JEG-3 magnification, 4x; decidual macrophages, 20x **(b)** DCFH-DA fluorescent intensities were quantified using CellProfiler software. Data are presented as the mean ± SD (n= 3 independent biological donors). Technical duplicates were performed for each donor and averaged before statistical analysis. Statistical analyses were performed using one-way ANOVA followed by Holm-Šídák’s multiple-comparisons test. Brackets denote the specific pairwise comparisons shown. Statistical significance is indicated as: ****p≤0.0001, ***p≤0.001, **p≤0.01, and *p≤0.05.

To determine whether melatonin influenced antioxidant response following oxidative stress, SOD2 mRNA expression was evaluated by qRT-PCR, and SOD2 enzymatic activity was assessed using a colorimetric assay (**Figure 5**). In JEG-3 cells, neither LPS stimulation nor melatonin treatment, regardless of treatment sequence, significantly altered SOD2 mRNA expression (**Figure 5a**). Likewise, SOD2 activity in JEG-3 cell lysates was below the assay detection limit and therefore was not further analyzed.

**Figure 5:**
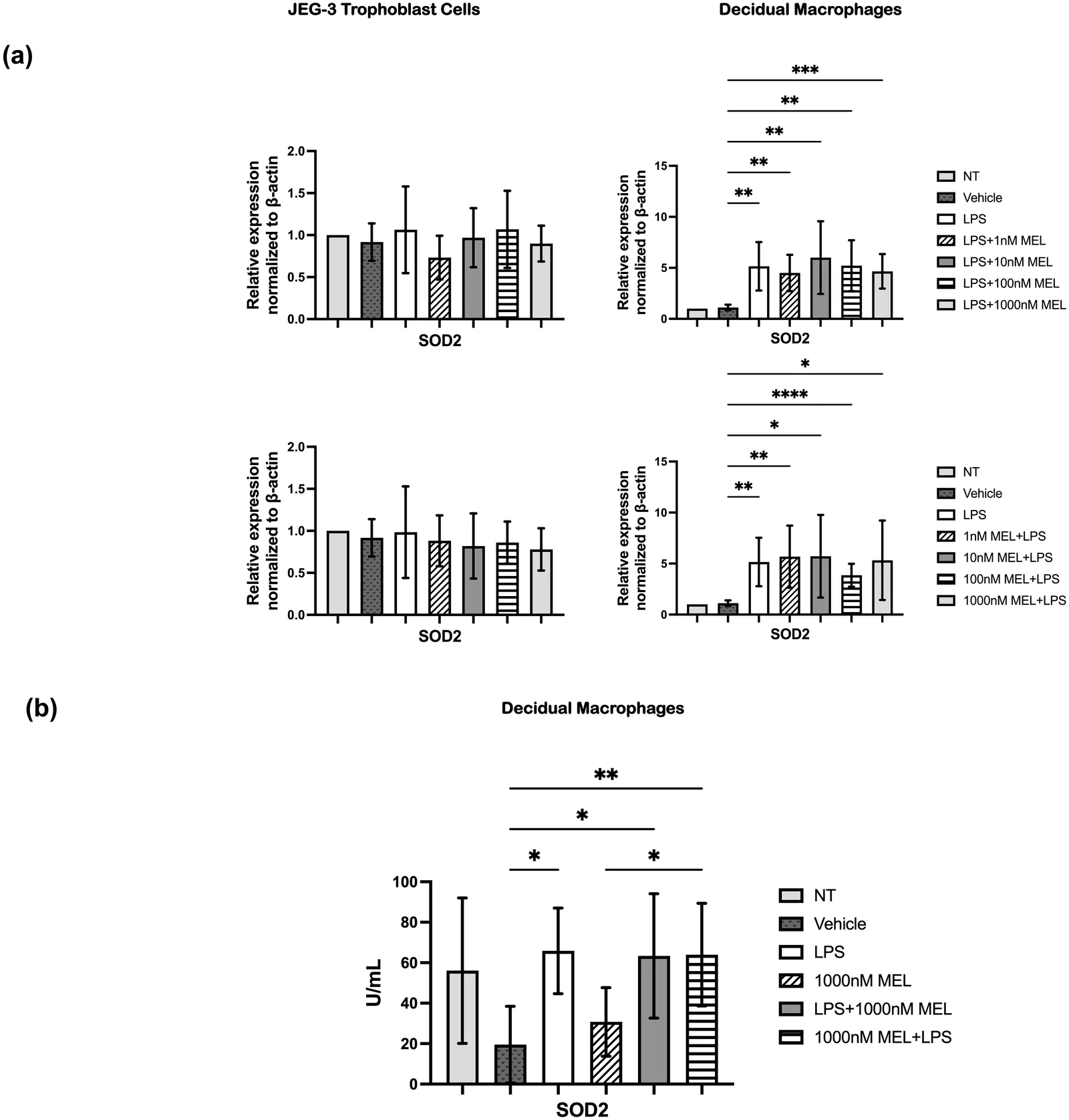
Melatonin increases SOD2 mRNA expression and activity in decidual macrophages. **(a)** JEG-3 trophoblast-like cells were cultured, and primary human decidual macrophages were isolated and treated with LPS (5 µg/mL), LPS for 4 h followed by melatonin (1–1000nM, 24 h), melatonin for 24 h followed by LPS for 4 h, or vehicle. SOD2 mRNA transcript levels were measured by qRT-PCR and expressed as ΔCT values normalized to β-actin. Data are presented as the mean ± SD (n= 5 independent biological donors). Technical duplicates were performed for each donor and averaged before statistical analysis. For JEG-3 data, statistical analyses were performed using the Kruskal–Wallis test followed by Dunn's multiple-comparisons test, whereas decidual macrophage data were analyzed using repeated-measures one-way ANOVA followed by Šídák's multiple-comparisons test. Brackets denote the specific pairwise comparisons shown. Statistical significance is indicated as: ****P ≤ 0.0001, ***P ≤ 0.001, **P ≤ 0.01, and *P ≤ 0.05. **(b)** Primary human decidual macrophages were isolated and treated with LPS (5 µg/mL), LPS for 4 h followed by melatonin (1000nM, 24 h), melatonin for 24 h followed by LPS for 4 h, melatonin for 24 h, or vehicle. SOD activity levels were determined using the Cayman Chemicals Superoxide Dismutase Assay kit according to the manufacturer’s instructions. Data are presented as the mean ± SD of four independent biological donors, with technical duplicates performed for each donor. For JEG-3 qRT-PCR data, statistical analyses were performed using the Kruskal–Wallis test followed by Dunn's multiple-comparisons test, whereas decidual macrophage data were analyzed using repeated-measures one-way ANOVA followed by Šídák's multiple-comparisons test. For SOD2 activity assay, statistical analyses were performed using Mixed-Effects followed by Šídák's multiple-comparisons test. Brackets denote the specific pairwise comparisons shown. Statistical significance is indicated as: ****P ≤ 0.0001, ***P ≤ 0.001, **P ≤ 0.01, and *P ≤ 0.05.

In primary decidual macrophages, LPS stimulation significantly increased SOD2 mRNA expression compared with the vehicle control. Elevated SOD2 transcript expression was maintained following melatonin treatment administered either before or after LPS stimulation relative to the vehicle control (**Figure 5a**). Assessment of SOD2 enzymatic activity demonstrated a similar pattern in decidual macrophages (**Figure 5b**). LPS significantly increased SOD2 activity compared with the vehicle control, while both post-and pre-treatment melatonin groups remained elevated relative to the vehicle. In addition, SOD2 activity was significantly increased in cells treated with 1000nM melatonin before LPS stimulation relative to the melatonin alone treatment group.

Collectively, these findings indicate that melatonin effectively reduced intracellular ROS in both placental cell types. In contrast, changes in SOD2 transcription and enzymatic activity were primarily observed in decidual macrophages, suggesting cell type-specific regulation of antioxidant responses during inflammatory responses.

### 3.7 Melatonin modulates oxidative stress-and inflammation-associated transcriptional programs in decidual macrophages

RNA seq-analysis was performed to characterize transcriptional changes in primary decidual macrophages treated with physiological (1nM) and supraphysiological (1000nM) melatonin. Compared with controls, 1nM melatonin (**Figure 6a**) increased transcripts associated with immune sensing and tolerance (*CASD1, CD180, PALD1, CD300LB, CDR2*, and *TGFBR1*), as well as metabolic and homeostatic processes (*FAM13A, WDR12*, and *GPAM*). In contrast, genes associated with oxidative stress responses, ROS production, and inflammatory activation (*SOD2, NCF1, VKORC1L1, IDO1*, and *DTX2*) were reduced, along with transcripts linked to cytoskeletal remodeling and intracellular communication (*GJB2, LIMK2*, and *MYO1G*).

**Figure 6:**
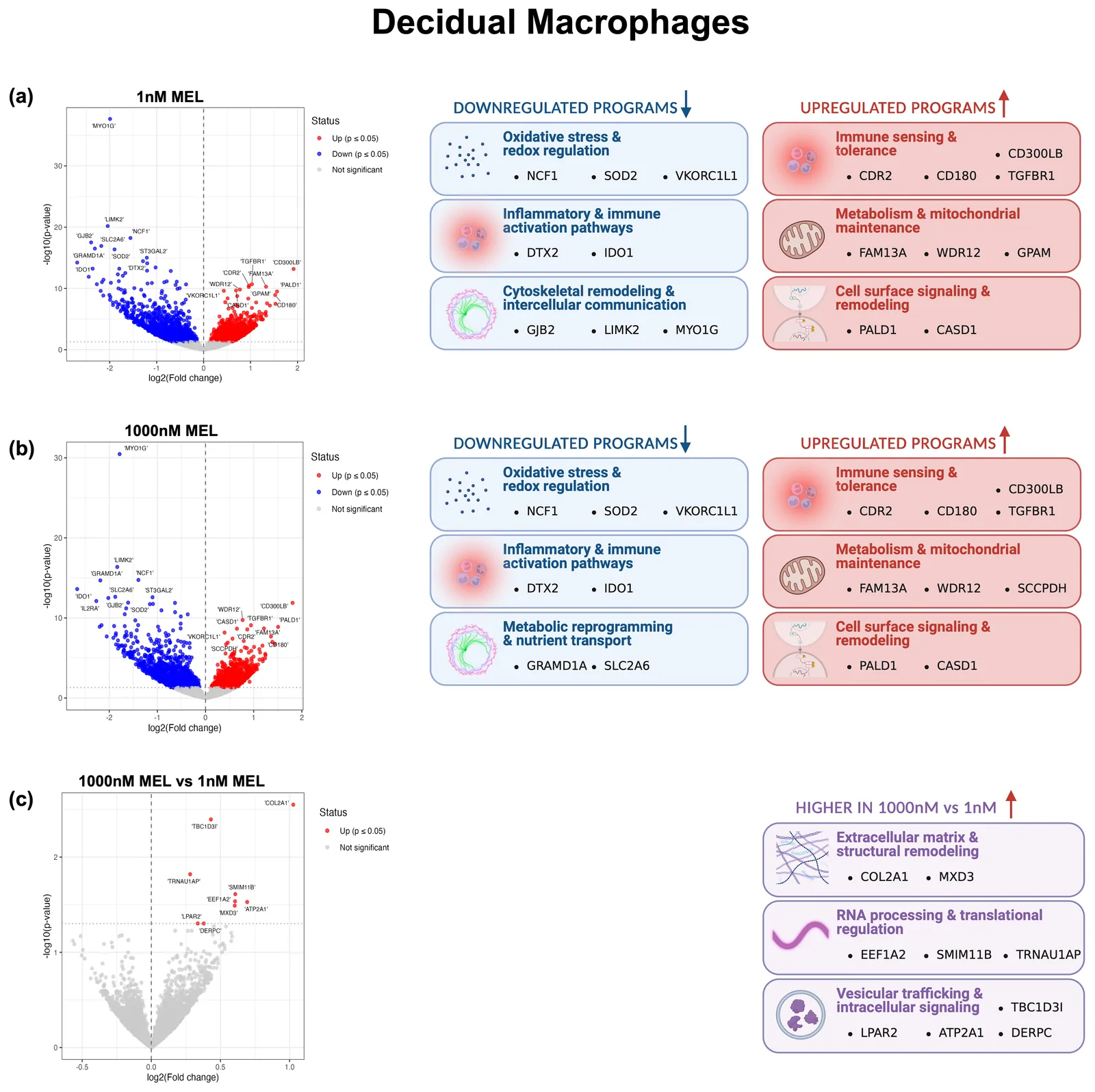
Transcriptomic profiling of human decidual macrophages following physiological and supraphysiological melatonin treatment. RNA-sequencing was performed on primary human decidual macrophages treated with vehicle, 1nM, or 1000nM melatonin. Differential gene expression analysis was performed using DESeq2, with significance defined as adjusted P (FDR) ≤ 0.05. **(a)** Volcano plot and corresponding gene tables showing differentially expressed genes (DEGs) in decidual macrophages treated with 1nM melatonin relative to vehicle-treated cells. Red dots represent significantly upregulated genes, blue dots represent significantly downregulated genes, and gray dots indicate non-significant transcripts. Gene tables summarize selected differentially expressed genes and their annotated cellular functions. **(b)** Volcano plot and corresponding gene tables showing DEGs in decidual macrophages treated with 1000nM melatonin relative to vehicle-treated cells. Red dots represent significantly upregulated genes, blue dots represent significantly downregulated genes, and gray dots indicate non-significant transcripts. Gene tables summarize selected differentially expressed genes and their annotated cellular functions. **(c)** Volcano plot comparing 1000nM versus 1nM melatonin-treated decidual macrophages. Red dots represent significantly upregulated genes, blue dots represent significantly downregulated genes, and gray dots indicate non-significant transcripts. Gene tables summarize selected differentially expressed genes and their annotated cellular functions.

At 1000nM melatonin (**Figure 6b**), a highly similar DEG profile was observed, with overlapping downregulation of oxidative and inflammatory transcripts and consistent suppression of cytoskeletal remodeling and metabolic processes. *SCCPDH* (cellular redox metabolism) replaced GPAM among the differentially expressed genes at 1000nM melatonin.

Direct comparison of 1000nM vs. 1nM melatonin (**Figure 6c**) identified a small set of upregulated genes (*COL2A1, TBC1D3I, TRNAU1AP, LPAR2, DERPC, MXD3, ATP2A1, SMIM11B*, and *EEF1A2*), and no significantly downregulated transcripts, indicating that both concentrations drive similar transcriptional outputs in decidual macrophages. Together, these findings indicate that melatonin was associated with altered expression of genes involved in oxidative stress responses, immune regulation, and cellular homeostasis in decidual macrophages under basal conditions. However, the functional consequences of these alterations during inflammatory challenge were not directly assessed in the RNA-seq experiments.

### 3.8 Melatonin induces modest transcriptomic changes in JEG-3-trophoblast-like cells

In JEG-3 cells, melatonin produced a comparatively modest transcriptional response relative to decidual macrophages. At 1nM (**Figure 7a**), only one transcript was significantly upregulated (*TPCN*, associated with calcium signaling and ER function). Several transcripts were downregulated, including TLR3 (viral-pattern recognition receptor), *ELOVL3* (lipid metabolism), and genes associated with developmental signaling, transcriptional regulation, and mitochondrial homeostasis (*PDGFRL, ELL3, CDC14C, LETM2*, and *SEMA3A*).

**Figure 7:**
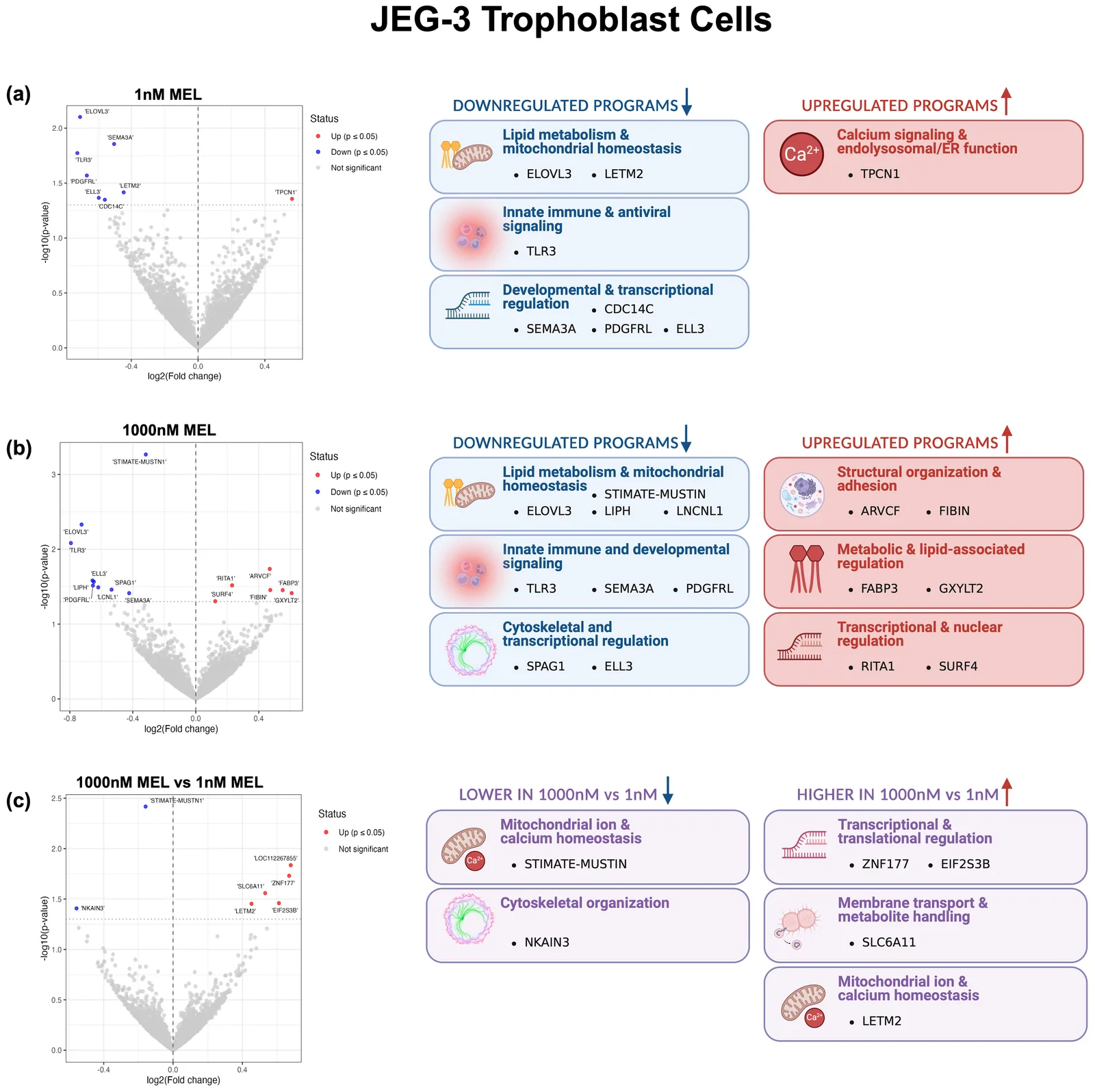
Transcriptomic profiling of JEG-3 trophoblast-like cells following physiological and supraphysiological melatonin exposure. RNA-sequencing was performed on JEG-3 trophoblast-like cells treated with vehicle, 1nM, or 1000nM melatonin. Differential gene expression analysis was performed using DESeq2, with significance defined as adjusted P (FDR) ≤ 0.05. **(a)** Volcano plot and corresponding gene tables showing differentially expressed genes (DEGs) in JEG-3 trophoblast-like cells treated with 1nM melatonin relative to vehicle-treated cells. Red dots represent significantly upregulated genes, blue dots represent significantly downregulated genes, and gray dots indicate non-significant transcripts. Gene tables summarize selected differentially expressed genes, their annotated cellular functions, and adjusted *P* values. **(b)** Volcano plot and corresponding gene tables showing DEGs in JEG-3 trophoblast-like cells treated with 1000nM melatonin relative to vehicle-treated cells. Red dots represent significantly upregulated genes, blue dots represent significantly downregulated genes, and gray dots indicate non-significant transcripts. Gene tables summarize selected differentially expressed genes, their annotated cellular functions, and adjusted *P* values. **(c)** Volcano plot comparing 1000nM and 1nM melatonin-treated JEG-3 trophoblast-like cells. Red dots represent significantly upregulated genes, blue dots represent significantly downregulated genes, and gray dots indicate non-significant transcripts. Gene tables summarize selected differentially expressed genes and their annotated cellular functions.

At 1000nM (**Figure 7b**), melatonin induced a slightly broader transcriptional response, with upregulation of genes associated with transcriptional regulation, membrane transport, and protein synthesis (*ZNF77, SLC6A11, EIF2S3B, LETM2*), while the downregulated gene set overlapped with the 1nM profile, with additional decreases in *STIMATE-MUSTN1* (mitochondrial function and calcium homeostasis). Direct comparison of 1000nM vs. 1nM melatonin (**Figure 7c**) revealed a similar transcriptional profile, with enrichment of the same upregulated gene set and continued downregulation of *NKAIN3* and *STIMATE-MUSTN1*, indicating minimal dose-dependent divergence between conditions These findings indicate that, under the experimental conditions examined, JEG-3 trophoblast-like cells exhibited comparatively limited transcriptional reprogramming in response to melatonin under basal conditions. The differentially expressed genes were primarily associated with calcium handling, membrane transport, mitochondrial function, cytoskeletal organization, and developmental signaling, with relatively limited representation of classical inflammatory and oxidative stress-associated genes.

### 3.9 Transcriptomic profiling revealed cell-specific transcriptional responses to melatonin at the maternal-fetal interface

Gene set enrichment analysis (FDR≤0.1) showed distinct, cell type-specific transcriptional responses to low (1nM) and high (1000nM) melatonin concentrations in both cell types (**Figure 8**). Pathways related to inflammation, oxidative stress, and cellular stress adaptation were significantly modulated, with both the magnitude and direction of changes varying by melatonin concentration and cell type. Immune-related pathways, including IL-6/JAK/STAT3 signaling, general inflammatory responses, and both type I and type II interferon pathways, were broadly negatively enriched in decidual macrophages at both melatonin concentrations, indicating reduced enrichment of gene sets associated with inflammatory signaling under basal treatment conditions. This effect was more pronounced at 1000nM, where nearly all inflammatory pathways exhibited strong negative enrichment, consistent with greater modulation of inflammatory transcriptional programs at the high melatonin concentration. In contrast, JEG-3 showed a more attenuated response. At 1nM, several inflammatory pathways were modestly negatively enriched, while at 1000nM, the response was more variable. While interferon and TNFα/NFκB signaling pathways remained negatively enriched, IL-6/JAK/STAT3 signaling showed neutral enrichment, indicating less pronounced negative enrichment of inflammatory pathways at the higher melatonin concentration. Overall, decidual macrophages exhibited a greater magnitude of transcriptional change across inflammatory pathways compared with JEG-3 cells, suggesting greater transcriptional responsiveness of maternal immune cells to melatonin treatment under basal conditions.

**Figure 8:**
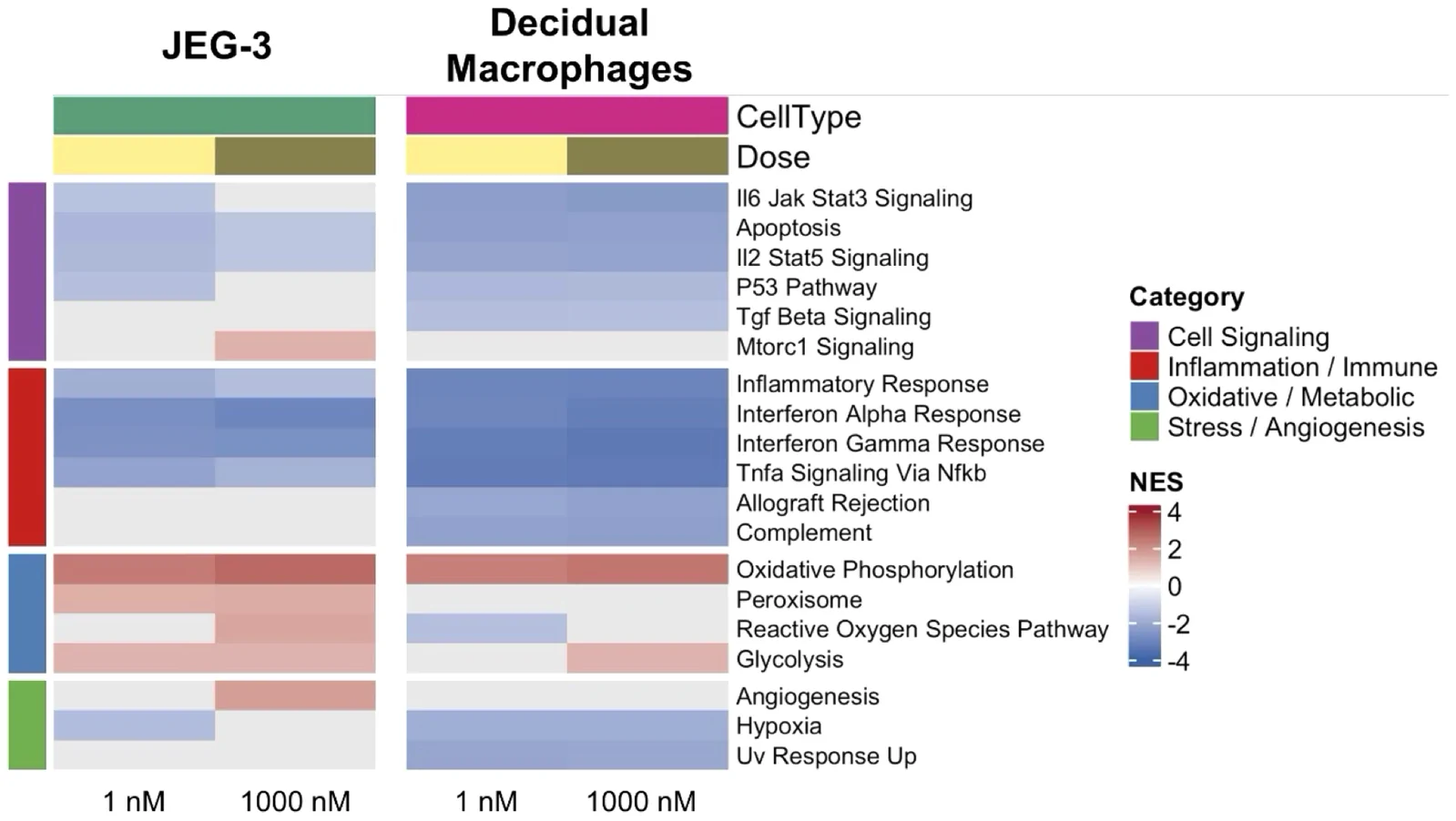
Transcriptomic profiling revealed melatonin-associated changes in inflammatory and oxidative stress- related pathways at the maternal-fetal interface. JEG-3 trophoblast-like cells and primary human decidual macrophages were treated with vehicle, 1nM melatonin, or 1000nM melatonin under basal conditions. Total RNA was extracted, and RNA sequencing was performed by BGI Genomics. Differential gene expression analysis was conducted using DESeq2 with median-ratio normalization and a negative binomial model. Gene set enrichment analysis (GSEA) was performed using fgsea with MSigDB Hallmark gene sets (category H). Genes were ranked according to the DESeq2 Wald statistic, and pathways were considered significantly enriched at FDR ≤ 0.10. Heatmaps depict normalized enrichment scores (NES), with red indicating positive enrichment and blue indicating negative enrichment across treatment conditions.

Melatonin also modulated pathways linked to oxidative stress and metabolic regulation, including oxidative phosphorylation, ROS-related signaling, peroxisome activity, hypoxia, p53, angiogenesis, and apoptosis. In decidual macrophages, oxidative phosphorylation was strongly positively enriched at both doses, and the ROS pathway enrichment was reduced at 1nM and partially reduces toward baseline at 1000nM, suggesting dynamic regulation of redox balance. Peroxisome and glycolytic pathways exhibited modest enrichment, consistent with metabolic adaptation rather than direct suppression of oxidative activity. In JEG-3 cells, oxidative phosphorylation and peroxisome pathways were also positively enriched at both doses, while ROS signaling showed minimal suppression at 1nM and increased enrichment at 1000nM. Additionally, pathways related to angiogenesis and hypoxia displayed variable responses, with evidence of increased enrichment at higher melatonin concentrations. These patterns suggest a more heterogeneous response in JEG-3 trophoblast-like cells compared with primary decidual macrophages.

Collectively, these findings suggest that melatonin induces stronger and more coordinated transcriptional reprogramming in decidual macrophages than in JEG-3 trophoblast-like cells. Decidual macrophages exhibit broader alterations in gene sets associated with inflammatory, oxidative stress, and metabolic pathways, whereas JEG-3 trophoblast-like cells demonstrated more modest and variable transcriptional responses. These results highlight the cell–type–dependent nature of melatonin signaling at the maternal–fetal interface and suggest that maternal immune cells may be more transcriptionally responsive to melatonin than JEG-3 trophoblast-like cells under basal conditions.

## 4 Discussion

A healthy pregnancy depends on a placenta that can maintain an appropriate balance between immune tolerance and protection (62). At the maternal-fetal interface, decidual macrophages and trophoblasts play complementary roles in maintaining this balance, creating a dynamic environment in which shifts in cellular homeostasis, oxidative stress, and inflammatory signals directly influence placental function (11, 63, 64). However, the mechanisms through which melatonin differentially regulates oxidative stress and inflammatory responses across placental cell types remain incompletely understood. In the present study, melatonin reduced LPS-induced cytokine secretion and intracellular ROS accumulation while modulating NRF2-associated responses, antioxidant defenses, and transcriptional programs in a cell type-specific manner. These effects were most pronounced in primary decidual macrophages, whereas JEG-3 trophoblast-like cells exhibited comparatively modest and treatment-sequence-dependent responses. Together, these findings are consistent with a model in which melatonin differentially regulates redox-inflammatory homeostasis at the maternal-fetal interface, with maternal immune cells demonstrating greater functional and transcriptional responsiveness than JEG-3 trophoblast-like cells during inflammatory stress.

A central finding of this study is that melatonin was associated with enhanced antioxidant responses and reduced oxidative stress in primary decidual macrophages. Melatonin was associated with increased NRF2 transcript expression under selected treatment conditions, reduced intracellular ROS accumulation, and increased SOD2 transcript expression and enzymatic activity under inflammatory conditions. These findings align with prior studies showing that melatonin stabilizes NRF2 by disrupting KEAP1-mediated ubiquitination, thereby promoting nuclear translocation and transcription of antioxidant response genes such as HO-1 and NQO1 (65). Although this mechanism has not been explicitly defined in decidual macrophages, similar NRF2 activation has been demonstrated in LPS-stimulated RAW264.7 macrophages and bone marrow–derived macrophages treated with melatonin, suggesting a similar regulatory response (66–68).

In parallel, melatonin reduced intracellular ROS accumulation and was associated with increased SOD2 transcript expression and enzymatic activity in decidual macrophages, further supporting its antioxidant capacity. Interestingly, although melatonin reduced intracellular ROS accumulation in both placental cell types, transcriptional and enzymatic antioxidant responses were pronounced in decidual macrophages, further supporting cell type-specific regulation of oxidative stress. Melatonin is an amphiphilic indoleamine that readily crosses cellular and mitochondrial membranes, where it can directly scavenge reactive oxygen and nitrogen species, reduce mitochondrial electron leakage, and stabilize mitochondrial function (24). These receptor-independent antioxidant effects are concentration-dependent and can occur rapidly, before transcriptional antioxidant programs are fully engaged. Prior studies demonstrate that melatonin can rapidly suppress mitochondrial ROS production independent of *de novo* transcription, allowing immune cells to achieve an antioxidant state more quickly than classical NRF2-driven responses alone (69).

Together, these findings suggest that the reduction in ROS observed in decidual macrophages may reflect both direct receptor-independent antioxidant activity and NRF2-associated antioxidant responses, although the relative contribution of these mechanisms was not directly examined in the present study. Collectively, these results suggest that melatonin supports antioxidant defenses in placental macrophages, which is highly relevant given that the placenta is exposed to dynamic inflammatory cues throughout pregnancy and must efficiently resolve oxidative stress to preserve tissue integrity and immune homeostasis (11, 42, 70).

In contrast, JEG-3 trophoblast-like cells responses to melatonin were more constrained. Although 1000nM melatonin increased pNRF2 protein expression under basal conditions in JEG-3 cells, this response was not maintained during inflammatory stimulation, and neither NRF2 transcript expression nor SOD2 expression was significantly altered following LPS treatment. Despite these limited transcriptional antioxidant responses, melatonin significantly reduced intracellular ROS accumulation, suggesting that antioxidant protection in JEG-3 trophoblast-like cells may occur through mechanisms that are not fully reflected by NRF2 transcription or SOD2 expression. This observation aligns with findings in epithelial cell models, where melatonin can activate NRF2 under LPS-induced oxidative stress; however, inflammatory signaling often dominates in regulating cellular responses (42, 71–73). Similarly, the comparatively modest transcriptomic changes observed in JEG-3 cells support the interpretation that melatonin elicits a more limited transcriptional response in JEG-3 trophoblast-like cells than in decidual macrophages.

Human trophoblasts synthesize melatonin locally and express melatonin receptors, supporting both autocrine/paracrine signaling and direct antioxidant protection within placental tissue (14). Because trophoblasts already possess strong intrinsic antioxidant defenses, exogenous melatonin may primarily act to stabilize mitochondrial redox balance rather than broadly altering gene transcription. Together, these data highlight the functional specialization of placental cell types at the maternal-fetal interface, with decidual macrophages exhibiting greater function and transcriptional responsiveness to melatonin during inflammatory stress, whereas JEG-3 trophoblast-like cells maintain a comparatively stable oxidative environment that may require less transcriptional adaptation (74).

Melatonin was also associated with changes in inflammatory gene expression in primary decidual macrophages. Although treatment-sequence-dependent differences in NLRP3 transcript expression were observed, these changes were not accompanied by significant alterations in total NLRP3 protein expression, and no significant differences were detected between LPS-treated cells and melatonin-treated groups. Previous studies have reported that melatonin modulates NLRP3-associated inflammatory responses in macrophages (42) and microglia (41). In the present study, IL-18 secretion was evaluated as an additional functional marker associated with inflammasome activation. Although IL-18 was detectable in primary decidual macrophages, no significant differences were observed among treatment groups, and IL-18 remained below the assay detection limit in JEG-3 trophoblast-like cells. Accordingly, the anti-inflammatory effects observed in decidual macrophages should not be interpreted as evidence of direct NLRP3 inflammasome regulation. Instead, these findings suggest that melatonin may influence broader inflammatory and oxidative stress responses under the experimental conditions examined. Future studies assessing caspase-1 activation, ASC oligomerization, and gasdermin D cleavage will be important for defining the contribution of NLRP3-associated inflammatory responses at the maternal-fetal interface.

The discordance observed between transcriptional and protein responses is not unexpected, as mRNA abundance does not necessarily correlate with protein expression. Protein levels are influenced by multiple post-transcriptional and post-translational mechanisms, including mRNA stability, translational efficiency, protein turnover, ubiquitin-mediated degradation, and differences in the temporal kinetics of transcription and protein accumulation. Moreover, activation of the NRF2 pathway is regulated largely through post-translational stabilization and nuclear translocation rather than transcription alone. Consequently, changes in transcript abundance may not be immediately reflected by corresponding changes in total protein levels under the experimental conditions examined.

Transcriptomic and pathway analysis further support these findings. Because transcriptomic profiling was performed under basal melatonin treatment conditions, these data should be interpreted as reflecting melatonin-responsive transcriptional programs rather than direct responses to inflammatory stimulation. In decidual macrophages, melatonin induced a coordinated transcriptional program characterized by negative enrichment of gene sets associated with IL-6/JAK/STAT3, interferon, and TNF-α/NFκB signaling, alongside modulation of oxidative phosphorylation and reactive oxygen species-associated pathways. These transcriptional patterns are consistent with previously described interactions between oxidative stress and inflammatory signaling pathways; however, because transcriptomic profiling was performed under basal conditions, the functional significance of these pathway changes during inflammatory stimulation remains to be determined (57). In addition, studies have shown that in dendritic cells exposed to LPS, melatonin reduced both oxidative stress and proinflammatory cytokines via the NRF2/HO-1 axis (68). In contrast, JEG-3 cells exhibited modest and variable transcriptional changes, with limited engagement of classical inflammatory pathways and greater emphasis on metabolic and structural processes. These findings suggest that decidual macrophages are more responsive to melatonin than JEG-3 trophoblast-like cells with respect to transcriptional programs associated with redox and inflammatory regulation, while JEG-3 trophoblast-like cells responses remain comparatively buffered. This greater transcriptional responsiveness closely paralleled the functional findings observed in decidual macrophages, including reduced cytokine secretion, diminished intracellular ROS accumulation, and enhanced SOD2 -associated antioxidant responses.

Differentially expressed gene analysis provides additional resolution on how melatonin shapes these responses at the gene level. In decidual macrophages, physiological and supraphysiological melatonin increased transcripts linked to immune sensing, tolerance, and cellular homeostasis, while reducing expression of genes associated with oxidative stress, ROS production, and inflammatory activation. This transcriptional profile is consistent with prior studies in macrophages, where melatonin dampens pro-oxidant and proinflammatory gene expression while promoting cytoprotective programs that support a controlled redox state (67, 75).

Interestingly, melatonin exhibited context-dependent effects. Under basal conditions, melatonin reduced expression of oxidative stress-related genes, whereas under LPS stimulation, antioxidant defenses such as SOD2 remained elevated. These findings are consistent with published data showing that melatonin preserves key antioxidant defenses during inflammatory stress, whereas under non-inflammatory conditions, it may reduce the need for elevated antioxidant gene expression by limiting upstream ROS pressure (76). Together, these findings suggest that melatonin differentially regulates antioxidant responses according to the cellular oxidative environment, by reducing transcriptional programs associated with oxidative stress under basal conditions while maintaining antioxidant defenses during inflammatory stimulation (26, 57, 67, 77–79).

In JEG-3 trophoblast-like cells, melatonin induced a comparatively modest DEG signature. At 1nM, only a small subset of genes was altered, including downregulation of TLR3 and other genes involved in lipid metabolism, signaling, and cellular structure. At 1000nM, a broader set of metabolic and structural genes was upregulated without strong engagement of classical inflammatory or oxidative stress networks. Together, these findings and the pathway analysis suggest that JEG-3 trophoblast-like cells primarily adjust metabolic and structural programs in response to melatonin, rather than mounting a robust anti-inflammatory or antioxidant transcriptional shift. This is consistent with prior work showing melatonin’s context-dependent modulation of mitochondrial, autophagy, and inflammatory programs in trophoblasts and other epithelial cells (30, 80). Collectively, these findings further support the greater transcriptional responsiveness of decidual macrophages to melatonin, whereas JEG-3 trophoblast-like cells exhibit more limited and pathway-specific transcriptional adaptations (41, 42, 81).

Although JEG-3 trophoblast-like cells are widely used as an *in vitro* placental model and retain several trophoblast-associated characteristics, including placental hormone production and responsiveness to inflammatory stimuli, they are derived from choriocarcinoma and therefore do not fully recapitulate the phenotype, metabolic activity or inflammatory responsiveness of primary trophoblast populations. Consequently, the comparatively modest inflammatory, antioxidant, and transcriptomic responses observed in JEG-3 trophoblast-like cells may reflect both the intrinsic biology of this experimental model and cell type-specific differences relative to primary decidual macrophages. Accordingly, these findings should not be interpreted as representative of all trophoblast populations, but rather as responses observed within this established trophoblast-like model under the experimental conditions examined. Validation in primary trophoblasts, placental explants, trophoblast organoids, and decidual-trophoblast co-culture systems will be important to determine the extent to which these observations translate to the intact maternal-fetal interface.

Dysregulated inflammation and oxidative stress contribute to multiple pregnancy complications, including preeclampsia, preterm birth, fetal growth restriction, and miscarriage (80, 81). The ability of melatonin to reduce LPS-induced cytokine release, influence NRF2-associated responses, reduce intracellular oxidative stress, and alter transcriptional pathways associated with inflammatory signaling in decidual macrophages suggests that melatonin may contribute to maintaining placental homeostasis during inflammatory stress. The cell-type-specific responses observed in the present study emphasize that the primary decidual macrophage and JEG-3 trophoblast-like cell models responded differently to melatonin under the experimental conditions examined. Primary decidual macrophages exhibited the most pronounced melatonin-associated changes across functional, cytokine, oxidative stress, and transcriptomic analyses, suggesting that maternal immune cells may represent an important cellular target of melatonin at the maternal-fetal interface. Given the central role of decidual macrophages in coordinating immune responses during pregnancy, modulation of these cells may influence placental inflammatory homeostasis. However, validation in primary trophoblasts, placental explants, trophoblast organoids, and co-culture models will be important to determine how these findings translate to the intact maternal-fetal interface.

In contrast, JEG-3 trophoblast-like cells responses were modest, consistent with their limited transcriptional responsiveness and intrinsic antioxidant capacity. Together, these findings suggest that trophoblasts may rely less on extensive transcriptional remodeling and more on constitutive antioxidant defenses under the experimental conditions examined. An additional finding of the present study was the treatment-sequence-dependent effect of melatonin. In decidual macrophages, melatonin pretreatment resulted in significantly lower IL-1β and IL-10 secretion than melatonin administered following LPS stimulation, whereas no significant difference was observed for IL-6. These findings indicate that the order of melatonin administration influenced select cytokine responses under the experimental conditions used. However, additional mechanistic studies are required to determine the biological basis of these treatment-sequence-dependent effects.

Several limitations should be considered when interpreting these findings. First, unstimulated melatonin-treated controls were not included in the qRT-PCR analyses performed under inflammatory conditions. Consequently, these experiments were designed to evaluate whether melatonin modified LPS-associated transcriptional responses rather than determine whether melatonin independently altered basal gene expression or restored transcript levels to unstimulated conditions. Accordingly, the qRT-PCR findings should be interpreted as demonstrating melatonin-associated modulation of inflammatory responses under the experimental conditions examined rather than restoration to basal expression levels. The transcriptomic analyses were performed under basal melatonin treatment conditions and therefore cannot directly determine how melatonin alters gene expression during LPS-induced inflammation. In addition, although melatonin treatment was associated with changes in NRF2-associated antioxidant responses, cytokine production, and oxidative stress, the molecular mechanisms linking these observations were not directly examined. Furthermore, canonical markers of NLRP3 inflammasome activation, including caspase-1 activation, ASC oligomerization, and gasdermin D cleavage, were not evaluated. Although IL-18 secretion was evaluated as a functional marker associated with inflammasome activation, no significant differences were observed among treatment groups in primary decidual macrophages, and IL-18 remained below the assay detection limit in JEG-3 trophoblast-like cells. Similarly, pNFκB and SOD2 protein expression were not assessed in the present study. In addition, an unexpected reduction in basal IL-1β secretion was observed in vehicle-treated JEG-3 cells compared with untreated controls. Because the present study was not designed to investigate vehicle-specific effects, the mechanism underlying this observation remains unclear. Although all treatment groups were interpreted relative to their corresponding vehicle controls, additional studies will be required to determine the reproducibility and biological significance of this vehicle-associated effect. Furthermore, the use of JEG-3-trophoblast-like cells represents an inherent limitation of the study, as this cell line does not fully recapitulate the complexity of primary trophoblast populations or the placental microenvironment *in vivo*. Despite these limitations, the combined functional and transcriptomic data provide insight into how melatonin influences inflammatory and oxidative stress responses in placental cells. Future studies incorporating primary placental cell populations, placental explants, *in vivo* pregnancy models, and targeted mechanistic approaches will help further define the pathways through which melatonin contributes to immune and redox regulation at the maternal-fetal interface.

In summary, our findings suggest that melatonin acts as a cell type-specific modulator of redox-inflammatory homeostasis at the maternal-fetal interface. Importantly, our data support a model in which melatonin may function through both receptor-independent antioxidant mechanisms and NRF2-associated antioxidant responses. In decidual macrophages, melatonin may reduce oxidative burden initially through direct radical scavenging and mitochondrial stabilization, followed by changes associated with NRF2-responsive antioxidant pathways. These complementary antioxidant mechanisms may collectively contribute to the reduced oxidative stress and inflammatory cytokine production observed in decidual macrophages during inflammatory stimulation. In contrast, JEG-3-trophoblast-like cells responses were comparatively modest, consistent with their limited transcriptional responsiveness and intrinsic antioxidant capacity. Interpreted in the context of placental physiology, these findings suggest that melatonin may contribute to maintain redox and immune homeostasis at the maternal-fetal interface by modulating redox and immune responses to preserve placental function during inflammatory stress. Disruption of this endogenous melatonin system, whether through reduced production, altered signaling, or overwhelming inflammatory burden, could increase susceptibility to placental injury. This vulnerability aligns with clinical patterns observed in preeclampsia, fetal growth restriction, miscarriage, and preterm birth, highlighting the broader significance of melatonin as a stabilizing factor in pregnancy.

## 5 Conclusion

Our findings show that melatonin influences inflammatory and oxidative stress pathways at the maternal-fetal interface in a cell type-specific manner. In decidual macrophages, melatonin was associated with reduced LPS-driven cytokine production and intracellular ROS accumulation while modulating NRF2-associated antioxidant responses. In contrast, JEG-3 trophoblast-like cells showed more modest responses, highlighting cell type-specific differences in melatonin responsiveness under the experimental conditions examined. Overall, these data suggest that melatonin was associated with attenuation of inflammatory signaling while supporting antioxidant responses in placental immune cells, with findings consistent with contributions from both receptor-independent antioxidant activity and NRF2-associated antioxidant responses. Given that dysregulated inflammation and oxidative stress contribute to complications such as preeclampsia, preterm birth, fetal growth restriction, and miscarriage, these findings suggest that melatonin may represent a potential adjunct strategy to modulate placental inflammation and preserve redox balance during inflammatory stress. Additional studies using primary trophoblasts, placental explants, trophoblast organoids, and *in vivo* pregnancy models are needed to validate these findings and determine the translational potential of melatonin in pregnancy.

## Data Availability

The datasets presented in this study can be found in online repositories. The names of the repository/repositories and accession numbers) can be found below: GSE3 13629 (GEO).
