# Supplementary material for: Melatonin is associated with cell-type-specific inflammatory and oxidative stress responses at the maternal-fetal interface
Source: Front Immunol. 2026 Sep 22;17:1897785. doi: 10.3389/fimmu.2026.1897785 (PMC13640950; doi:10.3389/fimmu.2026.1897785)
Supplement: Supplementary file 1 [file Table1.docx]

**S1 Table. Primer Sequences for qRT-PCR.**

| Gene | Forward Primer (5’-3’) | Reverse Primer (5’-3’) |
| --- | --- | --- |
| β-Actin | CACCATTGGCAATGAGCGGTTC | AGGTCTTTGCGGATGTCCACGT |
| NLRP3 | GGACTGAAGCACCTGTTGTGCA | TCCTGAGTCTCCCAAGGCATTC |
| NFκB | TGAACCGAAACTCTGGCAGCTG | CATCAGCTTGCGAAAAGGAGCC |
| NRF2 | CAGCGACGGAAAGAGTATGA | TGGGCAACCTGGGAGTAG |
| SOD2 | CTGGACAACCTCAGCCTAAC | AACCTGAGCCTTGGACACCAAC |

**S2 Table. Description of antibodies used in western blot experiments.**

| Antibody | Host | Provider | Catalog number / RRID | Dilution |
| --- | --- | --- | --- | --- |
| GAPDH | Rabbit | Cell Signaling Technology, Danvers, MA, USA | D16H11 | 1:1000 |
| NLRP3 | Rabbit | Cell Signaling Technology, Danvers, MA, USA | 13158S | 1:500 |
| NRF2 | Rabbit | Thermo Fisher Scientific, Waltham, MA, USA | PA5-27882 | 1:500 |
| pNRF2 | Rabbit | Bioss, Woburn, MA, USA | bs-23531R | 1:500 |
| Secondary Antibody | Goat | Cell Signaling Technology, Danvers, MA, USA | 7074S | 1:1000 |

**S3 Table. Description of antibodies used in immunofluorescence.**

| Antibody | Host | Provider | Catalog number / RRID | Dilution |
| --- | --- | --- | --- | --- |
| pNRF2 | Rabbit | Bioss, Woburn, MA, USA | bs-23531R | 1:500 |
| Secondary Antibody | Goat | Thermo Fisher Scientific, Waltham, MA, USA | A-11008 | 1:2500 |
| DAPI | N/A | Thermo Fisher Scientific, Waltham, MA, USA | D1306 | 1:1000 |

**S4 Table. Differentially expressed genes and functional classifications in decidual macrophages following melatonin treatment.**

**Lists of significantly upregulated and downregulated genes identified in decidual macrophages treated with 1nM or 1000nM melatonin compared with vehicle controls, as well as genes differentially expressed between 1000nM and 1nM melatonin conditions. Functional classifications were assigned based on biological relevance to immune regulation, oxidative stress, metabolism, mitochondrial function, and cellular signaling pathways.**

| **1nM MEL (Decidual Macrophages)**  **Provider** | | |
| --- | --- | --- |
| **Upregulated Genes** | | |
| **Genes** | **Cellular Function** | **p-value** |
| CD300LB | Immune Recognition & Innate Immune Modulation | 6.66E-14 |
| TGFBR1 | Immune Tolerance & Anti-inflammatory Signaling | 2.04E-11 |
| CDR2 | Immune Tolerance & Anti-inflammatory Signaling | 3.38E-11 |
| FAM13A | Lipid Metabolism & Mitochondrial/Energetic Homeostasis | 5.10E-11 |
| GPAM | Lipid Metabolism & Mitochondrial/Energetic Homeostasis | 5.75E-11 |
| WDR12 | Cellular Growth, Ribosome Biogenesis & Homeostatic Maintenance | 1.50E-10 |
| CASD1 | Glycosylation & Cell-Surface Modulation | 2.15E-10 |
| PALD1 | Vascular Signaling & Cellular Remodeling | 3.21E-10 |
| CD180 | Immune Recognition & Innate Immune Modulation | 1.18E-09 |
| **Downregulated Genes** | | |
| MYO1G | Cytoskeletal Dynamics & Cell Motility | 2.17E-38 |
| LIMK2 | Cytoskeletal Dynamics & Cell Motility | 6.64E-21 |
| NCF1 | Oxidative Stress & Redox Regulation | 5.83E-19 |
| GJB2 | Intracellular Communication | 3.08E-18 |
| SLC2A6 | Metabolic Reprogramming & Nutrient Transport | 1.17E-17 |
| GRAMD1A | Metabolic Reprogramming & Nutrient Transport | 3.11E-17 |
| SOD2 | Oxidative Stress & Redox Regulation | 4.26E-17 |
| ST3GAL2 | Glycosylation & Cell-Surface Signaling Modulation | 9.68E-16 |
| DTX2 | Inflammatory & Immune Activation Pathways | 3.50E-15 |
| IDO1 | Inflammatory & Immune Activation Pathways | 5.96E-15 |
| VKORC1L1 | Oxidative Stress & Redox Regulation | 2.40E-10 |

| **1000nM MEL (Decidual Macrophages)**  **Provider** | | |
| --- | --- | --- |
| **Upregulated Genes** | | |
| **Genes** | **Cellular Function** | **p-value** |
| CD300LB | Immune Recognition & Innate Immune Modulation | 6.66E-14 |
| TGFBR1 | Immune Tolerance & Anti-inflammatory Signaling | 2.04E-11 |
| CDR2 | Immune Tolerance & Anti-inflammatory Signaling | 3.38E-11 |
| FAM13A | Lipid Metabolism & Mitochondrial/Energetic Homeostasis | 5.10E-11 |
| WDR12 | Cellular Growth, Ribosome Biogenesis & Homeostatic Maintenance | 1.50E-10 |
| CASD1 | Glycosylation & Cell-Surface Modulation | 2.15E-10 |
| PALD1 | Vascular Signaling & Cellular Remodeling | 3.21E-10 |
| CD180 | Immune Recognition & Innate Immune Modulation | 1.18E-09 |
| SCCPDH | Mitochondrial Amino Acid Metabolism/ Cellular Redox Metabolism | 7.47E-08 |
| **Downregulated Genes** | | |
| MYO1G | Cytoskeletal Dynamics & Cell Motility | 2.17E-38 |
| LIMK2 | Cytoskeletal Dynamics & Cell Motility | 6.64E-21 |
| NCF1 | Oxidative Stress & Redox Regulation | 5.83E-19 |
| GJB2 | Intracellular Communication | 3.08E-18 |
| SLC2A6 | Metabolic Reprogramming & Nutrient Transport | 1.17E-17 |
| GRAMD1A | Metabolic Reprogramming & Nutrient Transport | 3.11E-17 |
| SOD2 | Oxidative Stress & Redox Regulation | 4.26E-17 |
| ST3GAL2 | Glycosylation & Cell-Surface Signaling Modulation | 9.68E-16 |
| IDO1 | Inflammatory & Immune Activation Pathways | 5.96E-15 |
| VKORC1L1 | Oxidative Stress & Redox Regulation | 2.40E-10 |

| **1000nM MEL VS 1nM MEL (Decidual Macrophages)**  **Provider** | | |
| --- | --- | --- |
| **Upregulated Genes** | | |
| **Genes** | **Cellular Function** | **p-value** |
| COL2A1 | Extracellular Matrix & Structural/Differentiation Programs | 2.82E-03 |
| TBC1D3I | Vesicular Trafficking & Intracellular Signal Modulation | 4.02E-03 |
| TRNAU1AP | Protein Translation & RNA Processing | 1.51E-02 |
| SMIM11B | Transcriptional & Cell-Cycle Regulation | 2.45E-02 |
| EEF1A2 | Protein Translation & RNA Processing | 2.91E-02 |
| ATP2A1 | Calcium Handling & ER Homeostasis | 2.97E-02 |
| MXD3 | Transcriptional & Cell-Cycle Regulation | 3.23E-02 |
| LPAR2 | GPCR, Lipid & Membrane Signaling | 4.97E-02 |
| DERPC | Vesicular Trafficking & Intracellular Signal Modulation | 4.98E-02 |

**S5 Table. Differentially expressed genes and functional classifications in JEG-3 trophoblast cells following melatonin treatment.**

**Lists of significantly upregulated and downregulated genes identified in trophoblast treated with 1nM or 1000nM melatonin compared with vehicle controls, as well as genes differentially expressed between 1000nM and 1nM melatonin conditions. Functional classifications were assigned based on biological relevance to immune regulation, metabolism and mitochondrial function, and cell regulation and maintenance.**

| **1nM MEL (Trophoblast Cells)**  **Provider** | | |
| --- | --- | --- |
| **Upregulated Genes** | | |
| **Genes** | **Cellular Function** | **p-value** |
| TPCN1 | Calcium Signaling & Endolysosomal/ER Function | 4.40E-02 |
| **Downregulated Genes** | | |
| ELOVL3 | Lipid Metabolism & Lipid Processing | 7.91E-03 |
| SEMA3A | Growth Factor & Developmental Signaling | 1.39E-02 |
| TLR3 | Innate Immune & Antiviral Sensing | 1.69E-02 |
| PDGFRL | Growth Factor & Developmental Signaling | 2.70E-02 |
| LETM2 | Mitochondrial Function & Ion/Calcium Homeostasis | 3.84E-02 |
| ELL3 | Transcriptional & Cell-Cycle Regulation | 4.30E-02 |
| CDC14C | Transcriptional & Cell-Cycle Regulation | 4.48E-02 |

| **1000nM MEL (Trophoblast Cells)**  **Provider** | | |
| --- | --- | --- |
| **Upregulated Genes** | | |
| **Genes** | **Cellular Function** | **p-value** |
| ARVCF | Cell Adhesion & Junctional Organization | 1.83E-02 |
| RITA1 | Transcriptional & Nuclear Regulation | 3.03E-02 |
| FIBIN | Developmental/Differentiation-Associated Signaling | 3.51E-02 |
| FABP3 | Lipid Binding & Fatty Acid Metabolism | 3.52E-02 |
| GXYLT2 | Glycosylation & Post-Translational Modification | 3.86E-02 |
| SURF4 | Transcriptional & Nuclear Regulation | 4.95E-02 |
| **Downregulated Genes** | | |
| STIMATE-MUSTIN1 | Mitochondrial Function & Ion/ Calcium Homeostasis | 5.41E-04 |
| ELOVL3 | Lipid Metabolism & Lipid Processing | 4.68E-03 |
| TLR3 | Innate Immune & Antiviral Signaling | 8.26E-03 |
| LIPH | Lipid Metabolism & Lipid Processing | 2.62E-02 |
| ELL3 | Transcriptional & Cell-Cycle Regulation | 2.72E-02 |
| PDGFRL | Growth Factor & Developmental Signaling | 3.05E-02 |
| LCNL1 | Lipid Metabolism & Lipid Processing | 3.23E-02 |
| SPAG1 | Cytoskeletal Organization, Motility & Structural Programs | 3.46E-02 |
| SEMA3A | Growth Factor & Developmental Signaling | 3.86E-02 |

| **1000nM MEL VS 1nM MEL (Trophoblast Cells)**  **Provider** | | |
| --- | --- | --- |
| **Upregulated Genes** | | |
| **Genes** | **Cellular Function** | **p-value** |
| LOC12267855 | Uncharacterized/ Predicted Gene Locus | 1.46E-02 |
| ZNF177 | Transcriptional Regulation | 1.86E-02 |
| SLC6A11 | Membrane Transport & Neurotransmitter/ Metabolite Handling | 2.76E-02 |
| EIF2S3B | Protein Translation & Biosynthetic Capacity | 3.47E-02 |
| LETM2 | Mitochondrial Function & Ion/ Calcium Homeostasis | 3.53E-02 |
| **Downregulated Genes** | | |
| STIMATE-MUSTIN1 | Mitochondrial Function & Ion/ Calcium Homeostasis | 3.82E-02 |
| NKAIN3 | Cytoskeletal Organization, Motility & Structural Programs | 3.91E-02 |

**S1 Figure. Cell viability following LPS and melatonin treatment in placental cells**

**Supplementary Figure S1.** Cell viability following treatment with LPS (5 µg/mL) and/or melatonin (1000nM). Cell viability was assessed using the MTT assay after 24 hours of treatment. Data are presented as the mean ± SD of **technical triplicates from one independent decidual macrophage donor and one independent JEG-3 cell experiment.** Statistical significance was determined using ordinary one-way ANOVA followed by Dunnett’s multiple comparison test. No significant differences in cell viability were observed among treatment groups.

**S2 Figure. Uncropped Western blot membranes corresponding to the NLRP3 immunoblots presented in Figure 1C.**

**Supplementary Figure S2.** Representative uncropped Western blot membranes for NLRP3 and the corresponding GAPDH loading controls from JEG-3 trophoblast-like cells and primary decidual macrophages are shown with molecular weight markers. Following protein transfer, PVDF membranes were horizontally sectioned at approximately the 50 kD molecular weight marker to permit independent probing of the upper membrane section for NLRP3 and the lower membrane section for GAPDH. Where applicable, membrane sections were sequentially stripped and reprobed with the indicated antibodies. Representative bands shown in the main manuscript were cropped for presentation only; all uncropped membranes are provided here for transparency.

**S3 Figure. Uncropped Western blot membranes corresponding to the NRF2 immunoblots presented in Figure 3b.**

**Supplementary Figure S3.** Representative uncropped Western blot membranes for NRF2 and the corresponding GAPDH loading controls from JEG-3 trophoblast-like cells and primary decidual macrophages are shown with molecular weight markers. Following protein transfer, PVDF membranes were horizontally sectioned at approximately the 50 kDa molecular weight marker to permit independent probing of the upper membrane section for NRF2 and the lower membrane section for GAPDH. Where applicable, membrane sections were sequentially stripped and reprobed with the indicated antibodies. Representative bands shown in the main manuscript were cropped for presentation only; all uncropped membranes are provided here for transparency.

**S4 Figure. Uncropped Western blot membranes corresponding to the NRF2 immunoblots presented in Figure 3b.**

**Supplementary Figure S4.** Representative uncropped Western blot membranes for pNRF2 and the corresponding GAPDH loading controls from JEG-3 trophoblast-like cells and primary decidual macrophages are shown with molecular weight markers. Following protein transfer, PVDF membranes were horizontally sectioned at approximately the 50 kDa molecular weight marker to permit independent probing of the upper membrane section for pNRF2 and the lower membrane section for GAPDH. Where applicable, membrane sections were sequentially stripped and reprobed with the indicated antibodies. Representative bands shown in the main manuscript were cropped for presentation only; all uncropped membranes are provided here for transparency.
